# Molecular‐Level Engineered Approach Induces Built‐in Electric Field Modulation in G‐C_3_N_4_/CoMoS_2_ Heterojunction for Enhanced Hydrogen Generation via Urea Oxidation

**DOI:** 10.1002/smll.73842

**Published:** 2026-05-19

**Authors:** Boka Fikadu Banti, Birhanu Bayissa Gicha, Mahendra Goddati, Hyojin Kang, Indra Memdi Khoris, Cheru Fekadu Molla, Sohrab Asgaran, Michael Giersig, Njemuwa Nwaji, Jaebeom Lee

**Affiliations:** ^1^ Department of Chemistry Chungnam National University Daejeon Republic of Korea; ^2^ Research Institute of Materials Chemistry Chungnam National University Daejeon South Korea; ^3^ Department of Chemical Engineering and Applied Chemistry Chungnam National University Daejeon Republic of Korea; ^4^ Institute For Sciences of the Universe Chungnam National University Daejeon Republic of Korea; ^5^ Helmaco Sp. Z o.o. Company Warszawa Poland; ^6^ Institute of Fundamental Technological Research Polish Academy of Sciences Warsaw Poland

**Keywords:** built‐in electric field, electrolysis, heterojunction, hydrogen production, urea oxidation

## Abstract

Urea‐assisted electrolysis boosts hydrogen production by substituting the sluggish oxygen evolution reaction (OER) with the energetically favorable urea oxidation reaction (UOR), thereby lowering energy consumption. Rational heterojunction engineering modulates charge distribution and generates abundant active sites, facilitating urea adsorption and C─N bond cleavage. Herein, we report a facile electrodeposition strategy to construct g‐C_3_N_4_/CoMoS_2_ hybrid electrocatalysts. The built‐in electric field at the heterojunction creates electrophilic regions on g‐C_3_N_4_ and nucleophilic regions on CoMoS_2_, selectively activating urea and promoting rapid bond cleavage. Anchoring g‐C_3_N_4_ onto CoMoS_2_ enables remarkable bifunctional activity toward both UOR and HER, achieving potentials of 1.27 V vs. RHE in 1 m KOH + 0.33 m urea and ‐80 mV vs. RHE in 1 m KOH at 10 mA cm^−2^, respectively. Density functional theory (DFT) calculations reveal that interfacial electron transfer enriches CoMoS_2_ with electrons and depletes g‐C_3_N_4_, enhancing charge transfer, optimizing urea adsorption, and lowering reaction energy barriers. Notably, the g‐C_3_N_4_/CoMoS_2_//g‐C_3_N_4_/CoMoS_2_ cell delivers 10 mA cm^−2^ at 1.34 V with excellent stability, demonstrating superior efficiency. This work provides a rational framework for designing efficient, energy‐saving urea‐assisted hydrogen production systems and reveals how intrinsic electric fields can precisely control charge distribution during catalysis.

## Graphical abstract

Urea‐assisted electrolysis is demonstrated as an energy‐efficient route for hydrogen production by replacing the sluggish oxygen evolution reaction with kinetically favorable urea oxidation. An intrinsic electric‐field‐driven g‐C_3_N_4_/CoMoS_2_ heterojunction induces directional charge redistribution, promotes selective urea activation, and accelerates C─N bond cleavage, enabling highly efficient and durable bifunctional electrocatalysis.

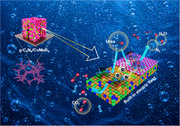

## 1 Introduction

Hydrogen (H_2_) is widely regarded as a promising carbon‐neutral energy carrier due to its high gravimetric energy density and environmental benignity [1, 2]. Electrocatalytic water splitting provides a sustainable route for H_2_ production; however, its efficiency is hindered by the sluggish kinetics and high overpotential (>1.23 V) of the anodic oxygen evolution reaction (OER), which leads to substantial energy losses [3, 4]. To overcome this limitation, replacing the OER with the anodic oxidation of small molecules such as methanol [5, 6, 7], 5‐hydroxymethylfurfural [8, 9], ethanol [10, 11, 12, 13], hydrazine [14, 15], and urea [16, 17, 18] has garnered significant attention as an energy‐efficient alternative. Among these, the urea oxidation is particularly attractive owing to its low theoretical potential (0.37 V vs. RHE), dual functionality in both energy generation and wastewater remediation, and natural abundance of urea [19, 20, 21, 22, 23]. Electrocatalysts capable of driving hydrogen production through the UOR hold significant promise for environmental cleanup, simultaneously enabling efficient energy conversion and pollutant removal [24, 25].

Notwithstanding this benefit, the six‐electron‐transfer nature of UOR engenders intrinsically sluggish kinetics, thereby necessitating the judicious conceptualization of catalysts that are both highly active and durable. Noble metal oxides such as RuO_2_ and IrO_2_ exhibit benchmark performance for both OER and UOR, but their high cost and scarcity limit large‐scale deployment [26, 27, 28]. As a result, research has focused on low‐cost, non‐noble‐metal catalysts capable of efficiently driving both UOR and the HER, offering simplified system designs with reduced energy and material requirements. Current design approaches for developing non‐precious metal catalysts are generally based on transition metals such as Ni, Co, Fe, etc., through the incorporation of non‐metallic elements, including oxygen [29], selenium [29], nitrogen [30, 31], phosphorus [32, 33], and sulfur [34], etc., to optimize catalytic activity. Emerging insights into the mechanism of UOR have resulted in guided design approaches such as heterostructure construction [35, 36, 37, 38], elemental doping [39, 40, 41], compositional regulation [42], defect engineering [43, 44, 45], doping‐induced reconstruction [46], and phase engineering [47], to boost electrocatalytic efficiency. The fundamental basis of electrocatalytic activity is surface adsorption‐desorption and electron‐transfer dynamics. This makes modulating the electronic structure of surfaces, particularly through heterojunction engineering, an effective strategy for enhancing catalytic performance [48]. Heterojunction interfaces induce lattice and electronic‐state modulation [35, 36], in which energy‐level offsets generate internal electric fields that redistribute charge and stabilize complementary electrophilic and nucleophilic sites [49]. For instance, Ni et al. [50] developed NiSe_2_/FeSe_2_ p–p heterojunctions, where the interfacial built‐in electric field accelerates charge transfer and induces local charge redistribution, enhancing intermediate adsorption and achieving 50 mA cm^−2^ at an overpotential of just 127 mV, surpassing both analogous materials and commercial RuO_2_. Similarly, He et al. [51] reported a heterojunction catalyst with enriched lattice defects and uniform interfaces, enhancing active site density, electron transfer, and surface area. In a UOR//HER system, it achieved 100 mA cm^−2^ at 1.453 V, 186 mV lower than the OER//HER system, while maintaining excellent durability in 1.0 m KOH/0.5 m urea. These studies highlight the effectiveness of heterojunction architectures in boosting UOR efficiency, although most fabrication methods rely on high temperatures or complex procedures.

Herein, we present a facile and cost‐effective approach for constructing a g‐C_3_N_4_/CoMoS_2_ heterojunction with a built‐in electric field through an environmentally friendly electrodeposition method. The electrode catalyst involves the in situ growth of a free‐standing CoMoS_2_ array on nickel foam (NF), followed by the anchoring of ultrathin g‐C_3_N_4_ nanosheets on the CoMoS_2_ arrays, forming an intimate heterointerface where the intrinsic band offset induces a spontaneous internal electric field. The spatial distribution of positive and negative charges is precisely modulated by the in‐built electric field at the heterojunction interface, thereby inducing the formation of electrophilic and nucleophilic domains. These positive and negative complementary regions exhibit strong adsorption toward the electron‐donating and electron‐withdrawing groups of urea molecules through electrostatic interactions, facilitating the cleavage of the C─N bond and the decomposition of urea.

First‐principles DFT calculations reveal work functions (𝚽) of 4.25 eV (g‐C_3_N_4_) and 5.11 eV (CoMoS_2_), confirming electron transfer from g‐C_3_N_4_ to CoMoS_2_ and validating the formation of an internal electric field that enhances both UOR and HER kinetics. These theoretical analyses provide key mechanistic insights and actionable design principles for tailoring interfacial properties. Collectively, the study establishes a robust framework for advancing urea‐assisted hydrogen generation with reduced energy requirements and improved catalytic efficacy, offering a viable pathway for next‐generation energy conversion systems.

## 2 Results and Discussion

### 2.1 Synthesis and Structural Characterization of G‐C_3_N_4_/CoMoS_2_ Heterojunction

**FIGURE 1:**
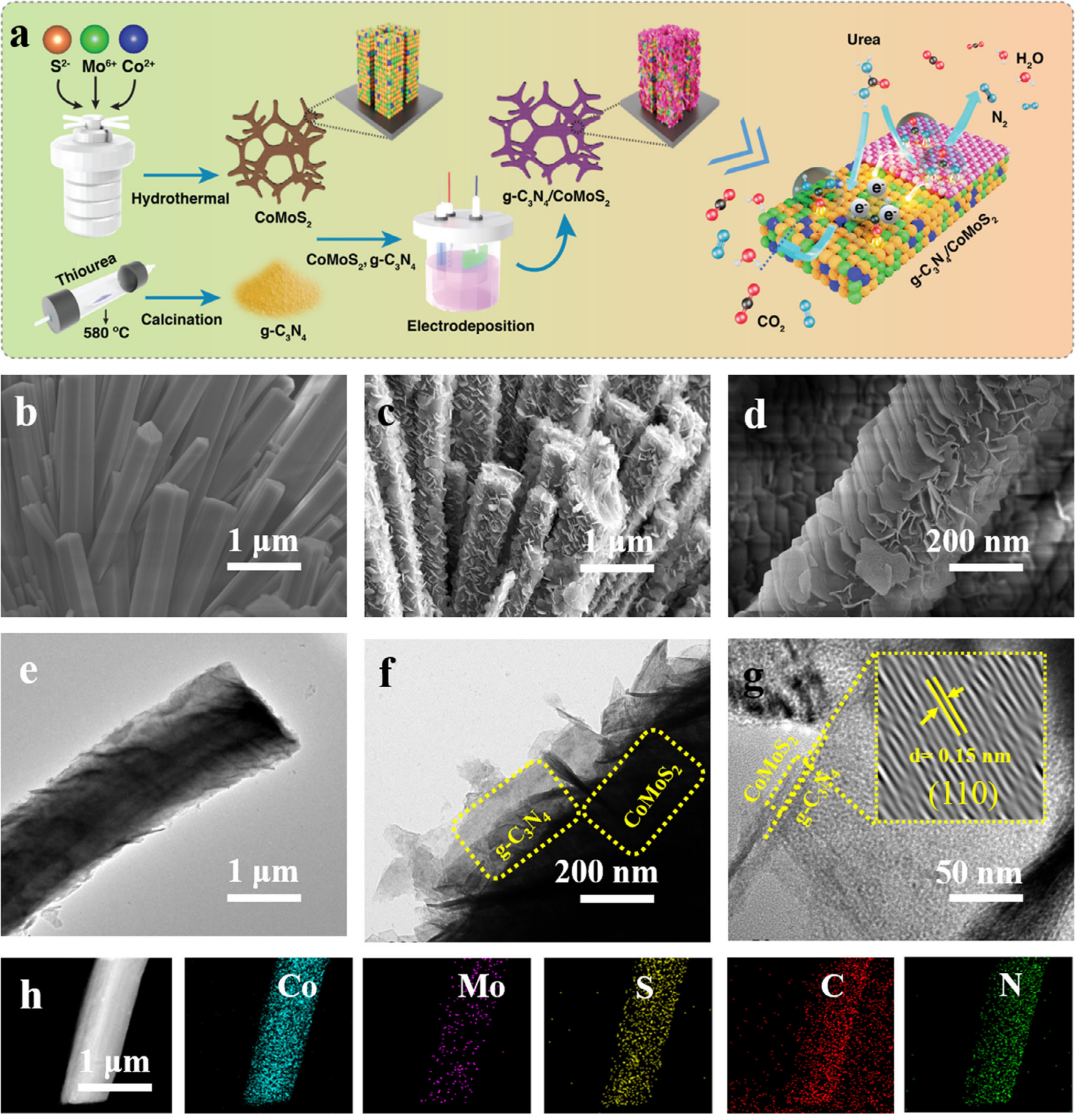
Synthesis and morphological characterization of the g‐C_3_N_4_/CoMoS_2_ interface catalyst. (a) Schematic representation of the fabrication process of the g‐C_3_N_4_/CoMoS_2_ catalyst. Representative SEM images of (b) CoMoS_2_ and (c,d) g‐C_3_N_4_/CoMoS_2_. (e,f) TEM images and (g) high‐resolution TEM (HR‐TEM) image of g‐C_3_N_4_/CoMoS_2_. (h) High‐angle annular dark‐field scanning TEM (HAADF‐STEM) image along with corresponding elemental mapping of the g‐C_3_N_4_/CoMoS_2_ catalyst, confirming the uniform distribution of constituent elements.

The g‐C_3_N_4_/CoMoS_2_ heterojunction was synthesized through a rational two‐step strategy, as illustrated in Figure 1a. Initially, graphitic carbon nitride (g‐C_3_N_4_) nanosheets (Figure S1) were prepared through direct thermal polymerization, while CoMoS_2_ nanorod arrays were grown in situ on nickel foam (NF) via a straightforward hydrothermal process. Subsequently, g‐C_3_N_4_ nanosheets were uniformly anchored onto the CoMoS_2_ nanorods through a potentiostatic electrodeposition, yielding the g‐C_3_N_4_/CoMoS_2_ heterojunction with significantly enhanced catalytic performance. The choice of electrodeposition arises from its simplicity, versatility, cost‐effectiveness, and efficiency. It is noteworthy that electrodeposition for one and two cycles results in incomplete nanorod coverage, whereas four cycles lead to pronounced aggregation (Figure S4), hindering electrolyte transport and compromising performance. Consequently, three electrodeposition cycles yield optimal performance. Detailed experimental procedures for catalyst synthesis are provided in the Experimental Section. The morphology of g‐C_3_N_4_/CoMoS_2_ and the individual components was systematically characterized by scanning electron microscopy (SEM) and transmission electron microscopy (TEM).

Figure 1b presents the SEM image of CoMoS_2_, revealing well‐defined, vertically oriented nanorod structures uniformly anchored to the NF substrate. Furthermore, Figure S2 presents SEM and TEM images at various magnifications, along with the corresponding elemental mapping of the CoMoS_2_ nanorod. As shown in Figure 1c,d and Figure S3a, the g‐C_3_N_4_/CoMoS_2_ heterojunction catalyst adopts a distinctive hierarchical architecture, with g‐C_3_N_4_ nanosheets uniformly decorating the vertically aligned CoMoS_2_ nanorods. This structural synergy leverages the complementary advantages of each component: the CoMoS_2_ nanorods serve as robust scaffolds and efficient electron transport channels, while the g‐C_3_N_4_ nanosheets, with their enlarged active surface area, enhance catalytic activity. The integration of g‐C_3_N_4_ nanosheets onto CoMoS_2_ nanorods effectively suppresses material aggregation and detachment, thereby supporting long‐term catalytic stability. TEM was employed to probe the fine structural features of the heterojunction. TEM images (Figure 1e,f and Figure S3b) show that the initially smooth CoMoS_2_ nanorods are uniformly coated with g‐C_3_N_4_ nanosheets. Fourier‐transformed high‐resolution TEM (**HRTEM**, Figure 1g) reveals clear lattice fringes with a spacing of 0.15 nm, corresponding to the (110) plane of CoMoS_2_, confirming the heterostructure's high crystallinity without noticeable structural distortions. This atomic‐level coupling at the g‐C_3_N_4_/CoMoS_2_ interface is expected to facilitate electronic communication and accelerate surface redox kinetics, enhancing electrocatalytic performance. High‐angle annular dark‐field scanning TEM (HAADF‐STEM) combined with elemental mapping (Figure 1g) demonstrates a homogeneous distribution of Co, Mo, S, C, and N throughout the heterojunction. Collectively, these results confirm the successful fabrication of g‐C_3_N_4_/CoMoS_2_ via interface engineering, forming a well‐defined heterojunction that expose abundant active sites, thereby boosting both HER and UOR performance [52].

**FIGURE 2:**
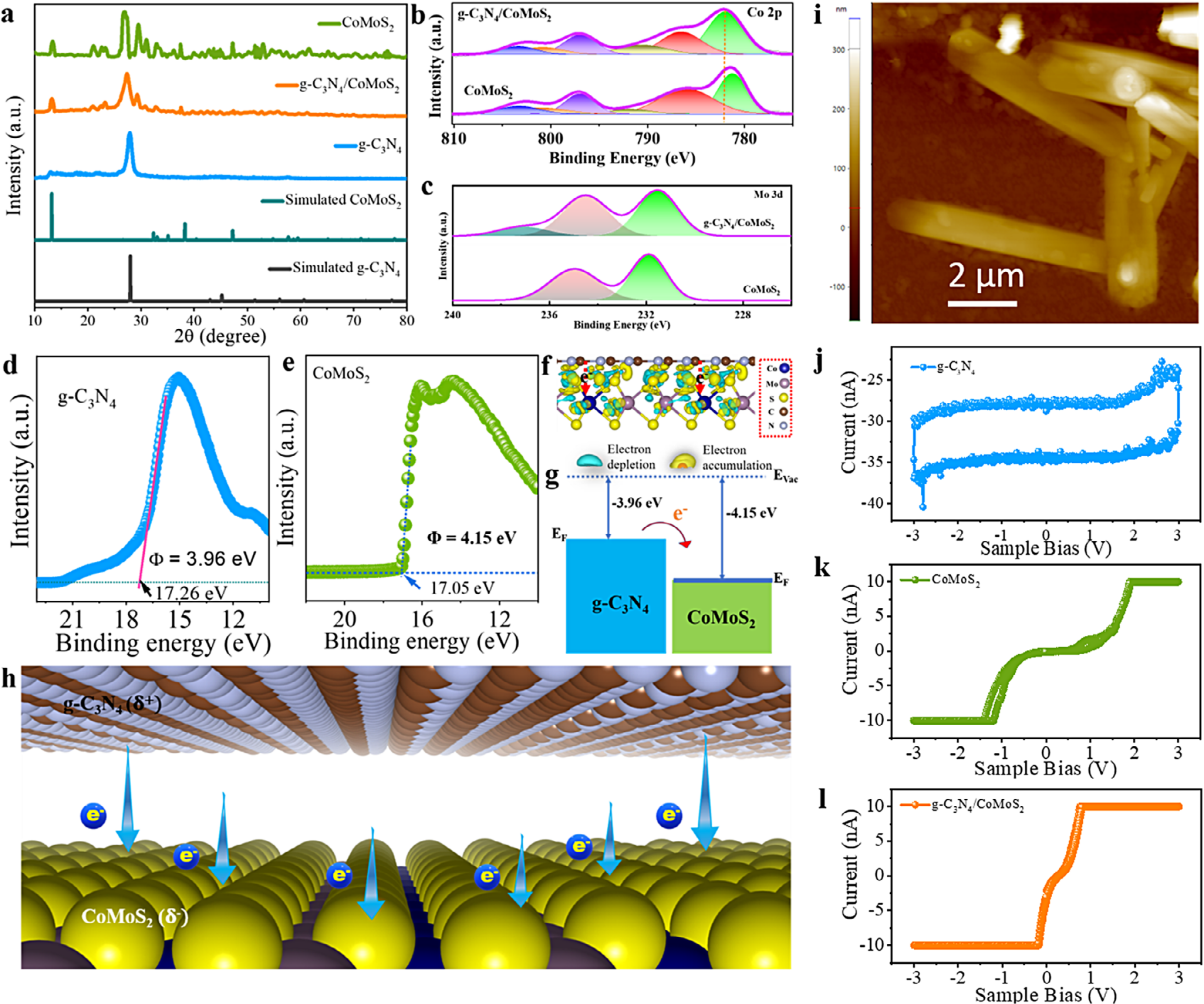
Crystallographic and structural characterization, together with Conductive Atomic Force Microscopy (C‐AFM) analysis, of the g‐C_3_N_4_/CoMoS_2_ interface and its associated charge‐transfer characteristics. (a) XRD patterns of g‐C_3_N_4_, CoMoS_2,_ and g‐C_3_N_4_/CoMoS_2_ catalysts. High‐resolution XPS spectra of (b) Co 2p and (c) Mo 3d. UPS spectra of (d) g‐C_3_N_4_ and (e) CoMoS_2_, showing their corresponding 𝚽. (f) Differential charge density of g‐C_3_N_4_/CoMoS_2_ heterojunction at equilibrium, with yellow and cyan isosurfaces (1.21 × 10^−3^ e bohr^−3^) indicating electron accumulation and depletion, respectively, revealing directional electron transfer from g‐C_3_N_4_ to CoMoS_2_. g) 𝚽 alignment between g‐C_3_N_4_ and CoMoS_2_. (h) Schematic of interfacial electron transfer between g‐C_3_N_4_ and CoMoS_2_. (i) Topographic Z‐height current image of g‐C_3_N_4_/CoMoS_2_. Current‐voltage curves using C‐AFM of (j) g‐C_3_N_4_, (k) CoMoS_2,_ and (l) g‐C_3_N_4_/CoMoS_2_ heterojunction.

X‐ray diffraction (XRD) analysis of g‐C_3_N_4_/CoMoS_2_ and the pristine components g‐C_3_N_4_ and CoMoS_2_ (Figure 2a) reveals well‐defined crystalline phases that are in excellent agreement with simulated structures, indicating high phase purity with negligible impurities. CoMoS_2_ exhibits diffraction peaks consistent with its simulated hexagonal phase, while g‐C_3_N_4_ shows a prominent (002) reflection at 2*θ* = 27.5°. The characteristic CoMoS_2_ peaks at 14.2°, 32.6°, and 58.3° correspond to the (002), (100), and (110) planes, confirming its layered hexagonal structure. The XRD patterns of the g‐C_3_N_4_/CoMoS_2_ heterojunction display the distinct reflections of both g‐C_3_N_4_ and CoMoS_2_, corroborating the successful formation of the heterojunction and the effective anchoring of g‐C_3_N_4_ nanosheets onto the CoMoS_2_ nanorod surface. X‐ray photoelectron spectroscopy (XPS) was employed to elucidate the surface composition, chemical states, and electronic configurations of g‐C_3_N_4_, CoMoS_2_, and their g‐C_3_N_4_/CoMoS_2_ heterojunction. The XPS survey spectra (Figure S5) show characteristic peaks corresponding to Co, Mo, S, N, and C, consistent with the elemental composition revealed by HAADF‐STEM analysis. The high‐resolution Co 2p XPS spectrum of CoMoS_2_ (Figure 2b) was deconvoluted into two doublet peaks at 781.4 and 797.6 eV, along with characteristic shake‐up satellite features. The first doublet peak at 781.4 and 786.3 eV can be assigned to Co^3+^, while the second doublet peak at 797.6 and 803.25 eV corresponds to Co^2+^, respectively [53, 54]. Relative to CoMoS_2_, the Co 2p peak in g‐C_3_N_4_/CoMoS_2_ exhibits a positive shift of 0.5 eV, indicative of pronounced electron redistribution at the g‐C_3_N_4_/CoMoS_2_ interface [55]. The Mo 3d XPS spectrum (Figure 2c) reveals a pair of peaks located at 231.4 and 234.5 eV corresponding to Mo 3d_5/2_ and Mo 3d_3/2_ doublets. The Mo 3d binding energy in g‐C_3_N_4_/CoMoS_2_ exhibits a negative shift of 0.4 eV relative to pristine CoMoS_2_, reflective of an increased local electron density. Likewise, the deconvoluted S 2p spectrum in CoMoS_2_ revealed a pair of peaks at binding energies of 160.95 and 162.10 eV, which can be assigned to the S 2p_3/2_ and S 2p_1/2_ of the Mo─S bonds (Figure S6). Compared to CoMoS_2_, the S‐2p of g‐C_3_N_4_/CoMoS_2_ shows a positive shift of approximately 0.3 eV (Figure S6), indicative of an increased local electron density. The observed change in binding energies following the formation of a heterojunction can be explained based on elemental interactions. In the g‐C_3_N_4_/CoMoS_2_ heterostructure, the nitrogen of g‐C_3_N_4_ serves as an electron donor, and the heterojunction interface acts as an electron acceptor. This synergistic effect optimizes a directional charge transfer channel, resulting in enhanced electron density accumulation around the Co─Mo─S bond, which will create a charge redistribution between Co, Mo and S. The observed positive shift in the binding energy of Co 2p and S 2p and negative shift for Mo 3d of the g‐C_3_N_4_/CoMoS_2_ relative to that of pristine CoMoS_2_ suggest electron deficiency Co and S and electron rich Mo, hence electrons are pushed toward the Mo. These XPS analyses unequivocally confirm the formation of a chemically coupled heterojunction and substantiate the presence of interfacial electronic synergy.

To quantitatively probe the textural architecture governing catalytic behavior, nitrogen adsorption‐desorption isotherms and Brunauer–Emmett–Teller (BET) analyses were performed to determine the specific surface area and pore size distribution of the materials (Figure S7). All catalysts display typical type IV isotherms accompanied by H3 hysteresis loops, indicative of well‐developed mesoporous architectures [56]. The intrinsic microporosity of g‐C_3_N_4_ anchored on CoMoS_2_ induces additional mesopores, resulting in a g‐C_3_N_4_/CoMoS_2_ heterojunction with a BET surface area more than three times higher than that of g‐C_3_N_4_ and CoMoS_2,_ as shown in Table S1. The construction of the heterojunction substantially enlarged the surface area and pore volume, underscoring that interface engineering is pivotal for electronic modulation and improved surface accessibility.

The interfacial charge‐transfer efficiency is intrinsically associated with the ease of electron extraction, which can be quantitatively described by the 𝚽 [57]. The 𝚽 of g‐C_3_N_4_ and CoMoS_2_ (Figure 2d,e) were determined from Ultraviolet Photoelectron Spectroscopy (UPS) using the equation

(1) $$\Phi =hv\;-\;\left(\;E_{cutoff}-E_{F}\right)$$

where Φ is the work function of the material, *h*νis the photon energy of the UV source (typically 21.22 eV for He I radiation), E_cutoff_ is the secondary electron cutoff, and E_F_​ is the Fermi level energy. Moreover, differential charge density analysis corroborates electron migration from g‐C_3_N_4_ to CoMoS_2_, leading to electron depletion on g‐C_3_N_4_ and accumulation on CoMoS_2_ (Figure 2f). Bader charge analysis indicates that approximately 1.64 e is transferred from g‐C_3_N_4_ to CoMoS_2_ across the g‐C_3_N_4_/CoMoS_2_ junction, demonstrating a strong degree of interfacial electronic coupling. Accordingly, the UPS analysis confirms that g‐C_3_N_4_ (3.96 eV) possesses a lower 𝚽 than CoMoS_2_ (4.15 eV), driving spontaneous electron transfer from g‐C_3_N_4_ to CoMoS_2_ upon contact. This charge redistribution continues until the Fermi levels equilibrate, thereby establishing a built‐in internal electric field across the heterointerface (Figure 2g,h). To gain microscopic insight into the enhanced electrocatalytic activity, conductive atomic force microscopy (C‐AFM) was employed to probe the local conductivity of the g‐C_3_N_4_/CoMoS_2_ heterojunction. Topographic Z‐height maps (Figure 2i) reveal that g‐C_3_N_4_ nanosheets uniformly decorate the vertically aligned CoMoS_2_ nanorods. Current mapping under forward and reverse bias further reveals distinct conduction characteristics among the materials. To isolate the intrinsic contribution of the g‐C_3_N_4_ component, current–voltage (*I*–*V*) characteristics were probed by conductive atomic force microscopy on pristine g‐C_3_N_4_ nanosheets (Figure 2j), revealing uniformly low currents across the entire bias window. *I*–*V* curves further reveal the distinct charge‐transport characteristics of pristine CoMoS_2_ (Figure 2k) compared with those of the g‐C_3_N_4_/CoMoS_2_ heterojunction (Figure 2l). The *I*–*V* characteristics of g‐C_3_N_4_/CoMoS_2_ heterojunction exhibit a steeper, more symmetric response than those of pristine CoMoS_2_, indicating enhanced charge injection and suppressed local hysteresis due to the formation of robust interfacial transport pathways.

### 2.2 Investigation of the Electrocatalytic Activity for the HER

**FIGURE 3:**
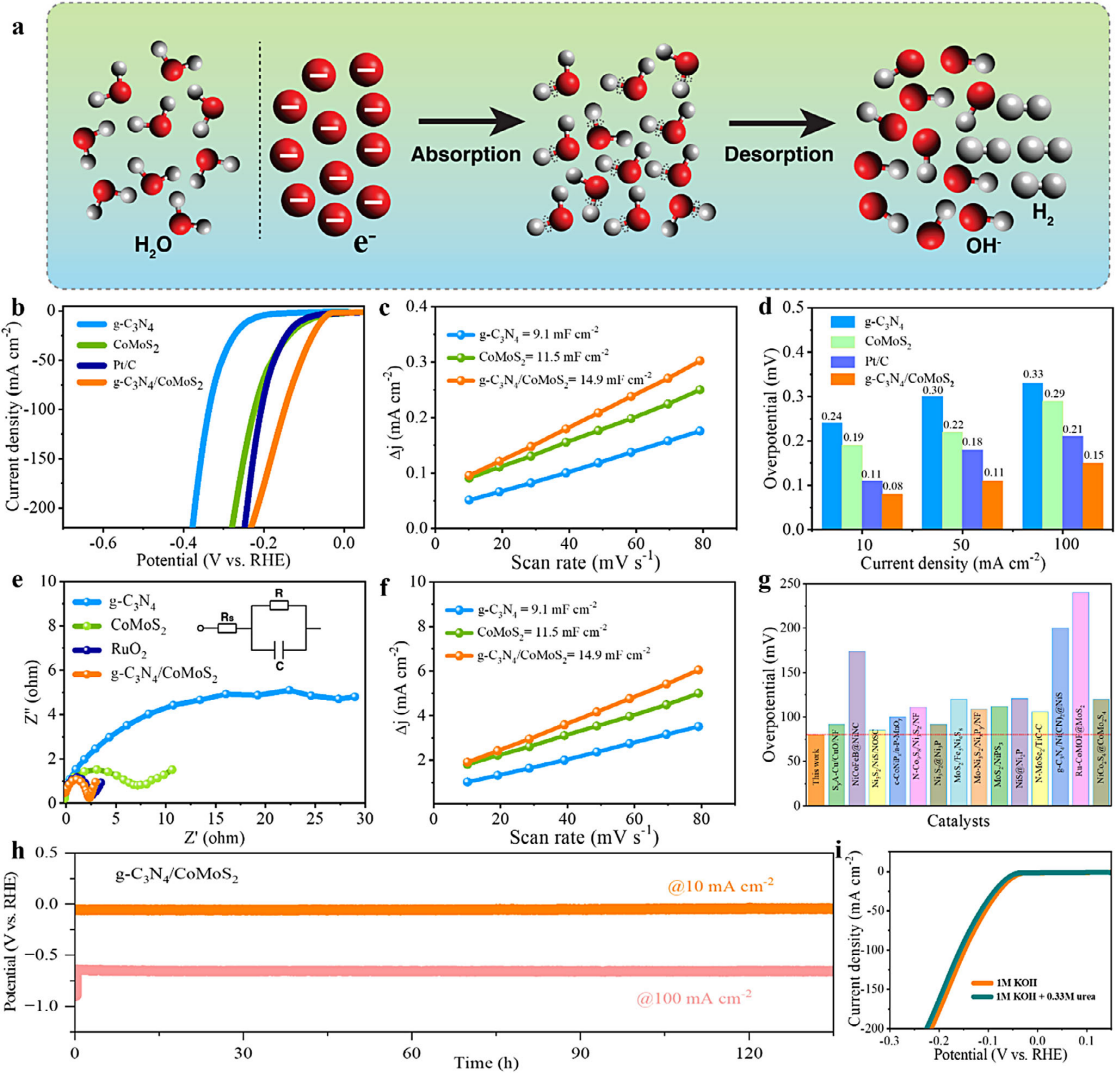
Electrocatalytic activity for the HER in 1.0 m KOH Solution. (a) Schematic illustration of the HER mechanism. (b) LSV curves; (c) Tafel slopes; (d) Overpotentials at 10, 50, and 100 mA cm^−2^; (e) Electrochemical impedance plots (Inset: equivalent circuit used in fitting the plot); (f) Calculated electrochemical double‐layer capacitance values of g‐C_3_N_4_, CoMoS_2_, Pt/C, and g‐C_3_N_4_/CoMoS_2_. (g) Comparison of the g‐C_3_N_4_/CoMoS_2_ catalyst with previously reported materials. (h) Chronopotentiometric stability test at 10 and 100 mA cm^−2^. (i) LSV curves of g‐C_3_N_4_/CoMoS_2_ in 1 m KOH electrolyte with and without 0.33 m urea.

To elucidate the structural advantages of g‐C_3_N_4_/CoMoS_2_ for HER, the electrocatalytic performance of g‐C_3_N_4_, CoMoS_2_, Pt/C, and g‐C_3_N_4_/CoMoS_2_ was systematically evaluated in 1.0 m KOH using a three‐electrode configuration. As schematically illustrated in Figure 3a, the alkaline HER proceeds through adsorption and dissociation of water molecules into adsorbed H^*^ intermediates on the catalytic surface (Volmer step), followed by their electrochemical desorption and H_2_ formation (Heyrovsky step). The linear sweep voltammetry (LSV) polarization curves (Figure 3b) demonstrate that g‐C_3_N_4_/CoMoS_2_ exhibits outstanding HER activity, requiring an overpotential of only 80 mV to achieve 10 mA cm^−2^, significantly lower than those of pristine g‐C_3_N_4_(280 mV), CoMoS_2_ (190 mV), and even commercial Pt/C (110 mV). To gain further insight into the intrinsic reaction kinetics, Tafel slopes derived from the corresponding polarization curves were analyzed (Figure 3c). The g‐C_3_N_4_/CoMoS_2_ heterojunction delivers a notably lower Tafel slope of 28.38 mV dec^−1^, compared to g‐C_3_N_4_ (62.43 mV dec^−1^), CoMoS_2_ (43.13 mV dec^−1^), and Pt/C (35.5 mV dec^−1^), confirming its accelerated reaction kinetics and indicating that the HER predominantly proceeds via the Volmer–Heyrovsky mechanism [58]. The overpotentials at various current densities (10, 50, and 100 mA cm^−2^) are summarized in Figure 3d, further confirming the superior catalytic efficiency of the heterojunction.

Electrochemical impedance spectroscopy (EIS) was employed to elucidate the charge‐transfer dynamics at the electrode‐electrolyte interface [59, 60]. As shown in Figure 3e, g‐C_3_N_4_/CoMoS_2_ exhibits a substantially reduced charge‐transfer resistance (R_ct_) compared to g‐C_3_N_4_, CoMoS_2,_ and Pt/C, indicating enhanced interfacial electron transport and superior catalytic kinetics. The electrochemically active surface area (ECSA) was further estimated by evaluating the double‐layer capacitance (C_dl_) from cyclic voltammetry in the non‐faradaic region (Figure S8). The g‐C_3_N_4_/CoMoS_2_ heterojunction exhibits an impressive C_dl_ value of 1.49 mF cm^−2^, surpassing that of g‐C_3_N_4_ (0.91 mF cm^−2^) and CoMoS_2_ (1.15 mF cm^−2^) (Figure 3f), implying a greater density of exposed active sites that facilitate enhanced hydrogen adsorption and accelerated HER kinetics. Furthermore, the g‐C_3_N_4_/CoMoS_2_ electrocatalyst demonstrates exceptional activity and durability, outperforming many recently reported non‐noble‐metal catalysts and ranking among the leading low‐overpotential, high‐stability systems (Figure 3g and Table S2). Long‐term chronopotentiometric measurements at 10 and 100 mA cm^−2^ (Figure 3h) reveal negligible potential drift over 135 h of continuous operation, attesting to the excellent structural integrity and chemical robustness of the heterojunction catalyst. The post‐test polarization curve (Figure S9) shows virtually no deviation from the initial measurement, confirming the outstanding durability and stability of the g‐C_3_N_4_/CoMoS_2_ heterojunction. Collectively, these comprehensive electrochemical evaluations affirm that the g‐C_3_N_4_/CoMoS_2_ heterojunction affords efficient charge transfer, abundant active sites, and durable catalytic interfaces. Finally, to establish continuity with preceding UOR studies, HER measurements were performed on g‐C_3_N_4_/CoMoS_2_ in 1 m KOH containing 0.33 m urea (Figure 3i). The negligible effect of urea on intrinsic HER activity indicates that, under practical operating conditions, UOR and HER can proceed simultaneously in a shared alkaline urea electrolyte, thereby simplifying system design and significantly reducing fabrication costs.

### 2.3 Electrocatalytic UOR Performance

**FIGURE 4:**
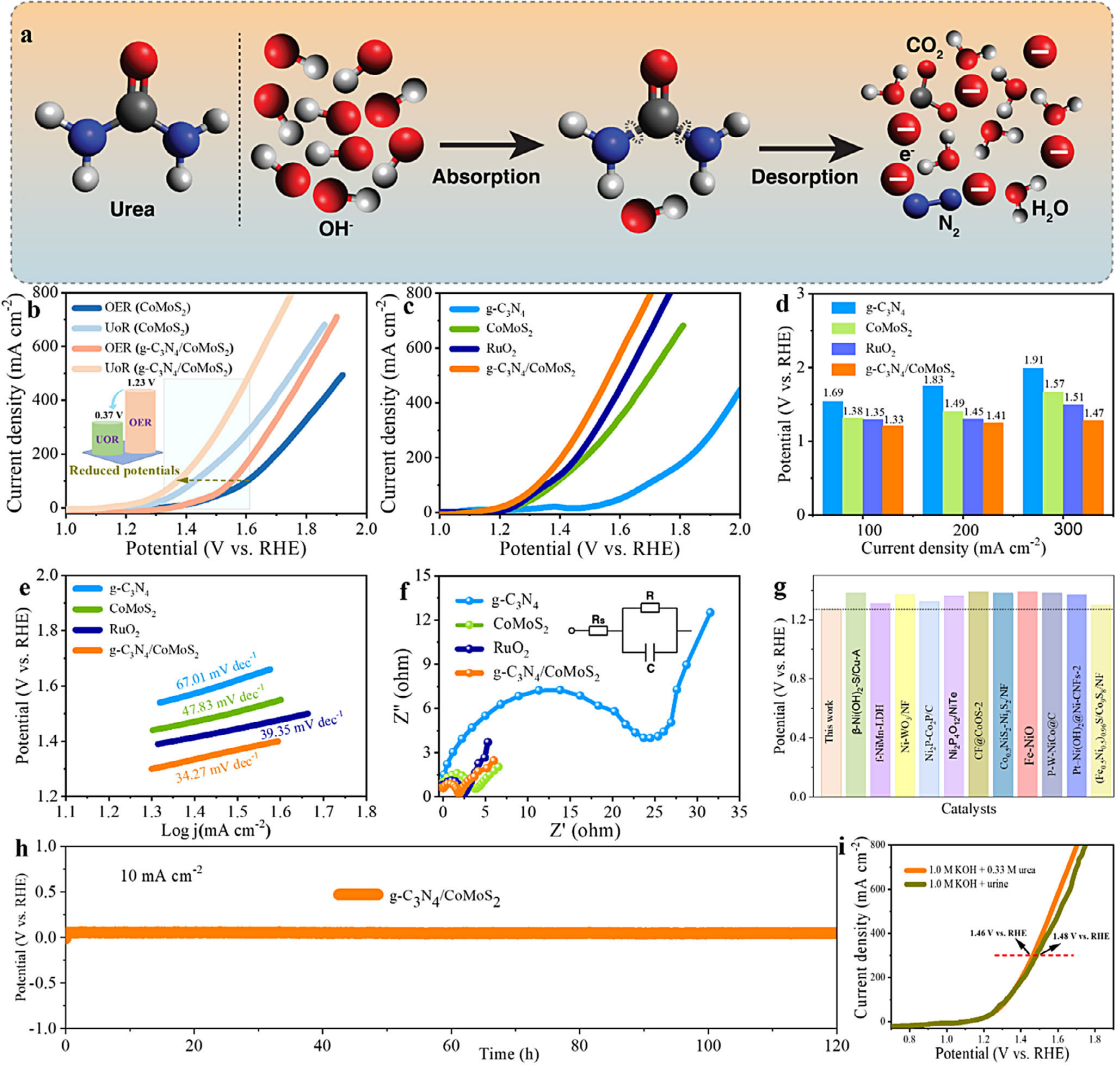
UOR electrocatalytic performance and stability. (a) Schematic illustration of the UOR process. (b) Comparison of OER and UOR LSV curves for CoMoS_2_ and g‐C_3_N_4_/CoMoS_2_ electrocatalysts. (c) LSV curves of UOR; (d) Potentials required to deliver current densities of 100, 200, 300 mA cm^−2^; (e) Corresponding Tafel slopes; (f) Electrochemical impedance plots of the as‐synthesized catalysts (Inset: equivalent circuit used in fitting the plot). (g) Comparison of the g‐C_3_N_4_/CoMoS_2_ catalyst with previously reported materials. (h) Chronopotentiometric stability test at 10 mA cm^−2^. (i) LSV curves of g‐C_3_N_4_/CoMoS_2_ for UOR in 1.0 m KOH containing urine.

Considering that the anodic reaction dictates the overall rate of water electrolysis, the synthesized materials were employed as UOR anodes and systematically evaluated in a standard three‐electrode setup at room temperature to elucidate their electrocatalytic behavior. As schematically depicted in Figure 4a, the UOR mechanism involves the initial adsorption of urea molecules on the catalyst surface, followed by their dissociation into key intermediates (^*^CO, ^*^COOH, and ^*^NH), wherein this dissociation step acts as the rate‐determining process and consequently governs the overall efficiency of urea electrooxidation. The UOR performance of the g‐C_3_N_4_/CoMoS_2_ heterojunction was first evaluated in 1.0 m KOH under changing urea concentrations to uncover the concentration‐dependent catalytic behavior. As presented in Figure S10, the well‐defined g‐C_3_N_4_/CoMoS_2_ heterojunction efficiently catalyzes urea oxidation in alkaline media. The anodic current density exhibits a pronounced increase with rising urea concentration from 0.1 to 0.33 m, followed by a gradual decline at higher concentrations. This decrease is attributed to excessive urea molecules that hinder the accessibility of OH^−^ near the active sites, thereby suppressing C─N bond cleavage and limiting the kinetics of UOR. LSV was performed to probe the competitive dynamics of urea oxidation and oxygen evolution in 1.0 m KOH, with and without 0.33 m urea. As depicted in Figure 4b, introducing urea markedly amplifies the anodic current density compared with OER and induces a clear leftward shift of the polarization curve. Specifically, g‐C_3_N_4_/CoMoS_2_ and CoMoS_2_ require only 1.27 and 1.31 V, respectively, to achieve 10 mA cm^−2^, whereas the corresponding OER potentials increase to 1.47 and 1.52 V. Figure 4c compares the LSV curves for g‐C_3_N_4_/CoMoS_2_, g‐C_3_N_4_, CoMoS_2_, and the benchmark RuO_2_ in 1.0 m KOH containing 0.33 m urea, while OER measurements were conducted in 1 m KOH without urea (Figure S11). Notably, g‐C_3_N_4_/CoMoS_2_ exhibits the most pronounced UOR activity, delivering the lowest potential among all catalysts. Furthermore, this heterojunction requires significantly reduced overpotentials to achieve high current densities (100, 200, and 300 mA cm^−2^) relative to the pristine components and RuO_2_ (Figure 4d). Kinetic analysis based on Tafel slopes (Figure 4e) reveals that g‐C_3_N_4_/CoMoS_2_ heterojunction exhibits a markedly smaller slope (34.27 mV dec^−1^) compared with g‐C_3_N_4_ (67.01 mV dec^−1^), CoMoS_2_ (47.83 mV dec^−^^1^), and RuO_2_ (39.35 mV dec^−^^1^), indicating more favorable reaction kinetics. EIS further confirms the enhanced charge‐transfer properties of the heterojunction. As depicted in Figure 4f, g‐C_3_N_4_/CoMoS_2_ exhibits a significantly reduced charge‐transfer resistance (R_ct_), evidencing accelerated electron transport at the electrode/electrolyte interface.

As summarized in Figure 4g and Table S3, the g‐C_3_N_4_/CoMoS_2_ catalyst outperforms most recently reported UOR electrocatalysts. Beyond catalytic activity, long‐term operational durability, a critical metric for industrial applicability, was assessed via chronopotentiometry. The g‐C_3_N_4_/CoMoS_2_ heterojunction exhibits outstanding stability, maintaining a constant potential with negligible activity loss over 120 h of continuous operation at 10 mA cm^−2^ (Figure 4h). The nearly overlapping LSV curves before and after the durability test (Figure S12) confirm the material's excellent electrochemical stability in 1 m KOH with 0.33 m urea. To elucidate the structural robustness of the catalyst, post‐UOR characterizations, including XRD, XPS, and SEM (Figures S13–S15), were performed on g‐C_3_N_4_/CoMoS_2_. The consistent morphological, crystallographic, and surface chemical features observed after prolonged electrolysis confirm that the g‐C_3_N_4_/CoMoS_2_ heterojunction preserves its structural integrity and interfacial stability under extended operational conditions. Given the abundance of urea in domestic and industrial effluents, the electrocatalytic performance of g‐C_3_N_4_/CoMoS_2_ was assessed under practical conditions using human urine in 1.0 m KOH as the electrolyte for urea oxidation. As shown in Figure 4i, the catalyst achieves a current density of 300 mA cm^−2^ at a relatively low potential of 1.48 V vs. RHE, highlighting its outstanding activity in real urine. Compared with its performance in a standard 1.0 m KOH + 0.33 m urea electrolyte (1.46 V vs. RHE), the working potential in urine is slightly elevated, likely due to the presence of inorganic salts and other impurities, which can impede charge transfer and modestly reduce overall UOR efficiency. Stability was further evaluated under practical conditions using KOH with human urine as the electrolyte. The g‐C_3_N_4_/CoMoS_2_ heterojunction sustains a stable potential at 100 mA cm^−2^ over 120 h with negligible performance decay (Figure S16), underscoring its remarkable electrochemical resilience. Such stability reflects a pronounced resistance to chloride‐induced corrosion and a high tolerance toward complex organic constituents in urine. This robustness is ascribed to the well‐integrated heterointerface and finely tuned electronic coupling, which collectively preserve active site integrity and mitigate surface poisoning.

**SCHEME 1:**
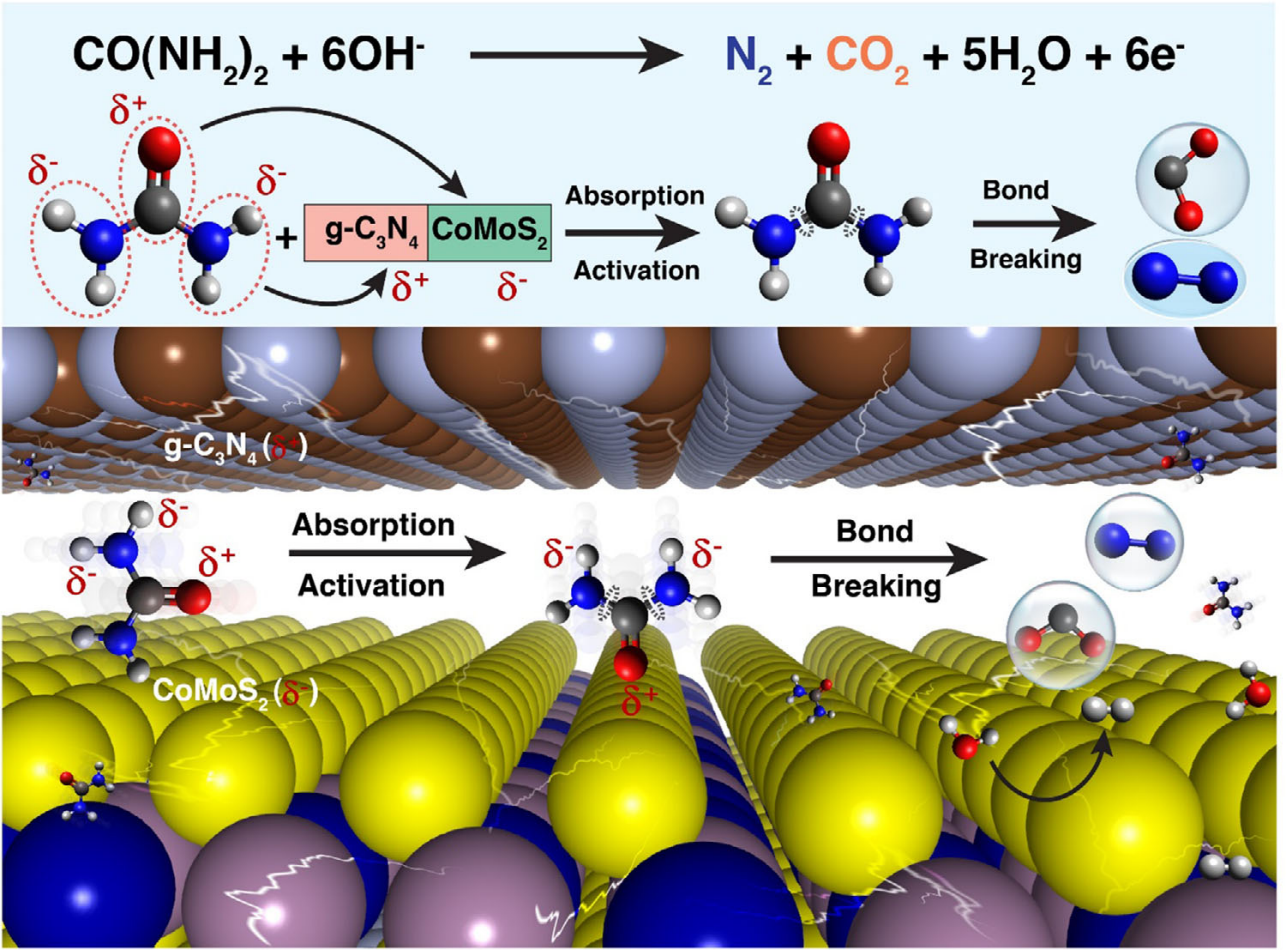
Schematic of the proposed UOR mechanism at the g‐C_3_N_4_/CoMoS_2_ interface, indicating cooperative adsorption, electronic activation, and C─N bond cleavage to produce N_2_ and CO_2_.

In view of the above results, Scheme 1 provides a schematic depiction of the potential urea electrooxidation process on the g‐C_3_N_4_/CoMoS_2_ heterojunction surface. The spontaneous electron redistribution from g‐C_3_N_4_ to CoMoS_2_ generates an internal interfacial electric field, leading to electron‐deficient (electrophilic) regions on g‐C_3_N_4_ and electron‐rich (nucleophilic) regions on CoMoS_2_. During UOR, the electron‐rich amino group of urea preferentially coordinates with the electrophilic sites of g‐C_3_N_4_, while the electron‐withdrawing carbonyl group interacts with the nucleophilic domains of CoMoS_2_, guided by interfacial electrostatic interactions. This cooperative electronic modulation across the heterointerface facilitates the activation and cleavage of the C─N bonds. In parallel, the lower 𝚽 of g‐C_3_N_4_ within the g‐C_3_N_4_/CoMoS_2_ architecture promotes the rapid desorption of reaction intermediates, thereby further enhancing the kinetics of urea electrooxidation.

### 2.4 In Situ Raman Spectroscopy

**FIGURE 5:**
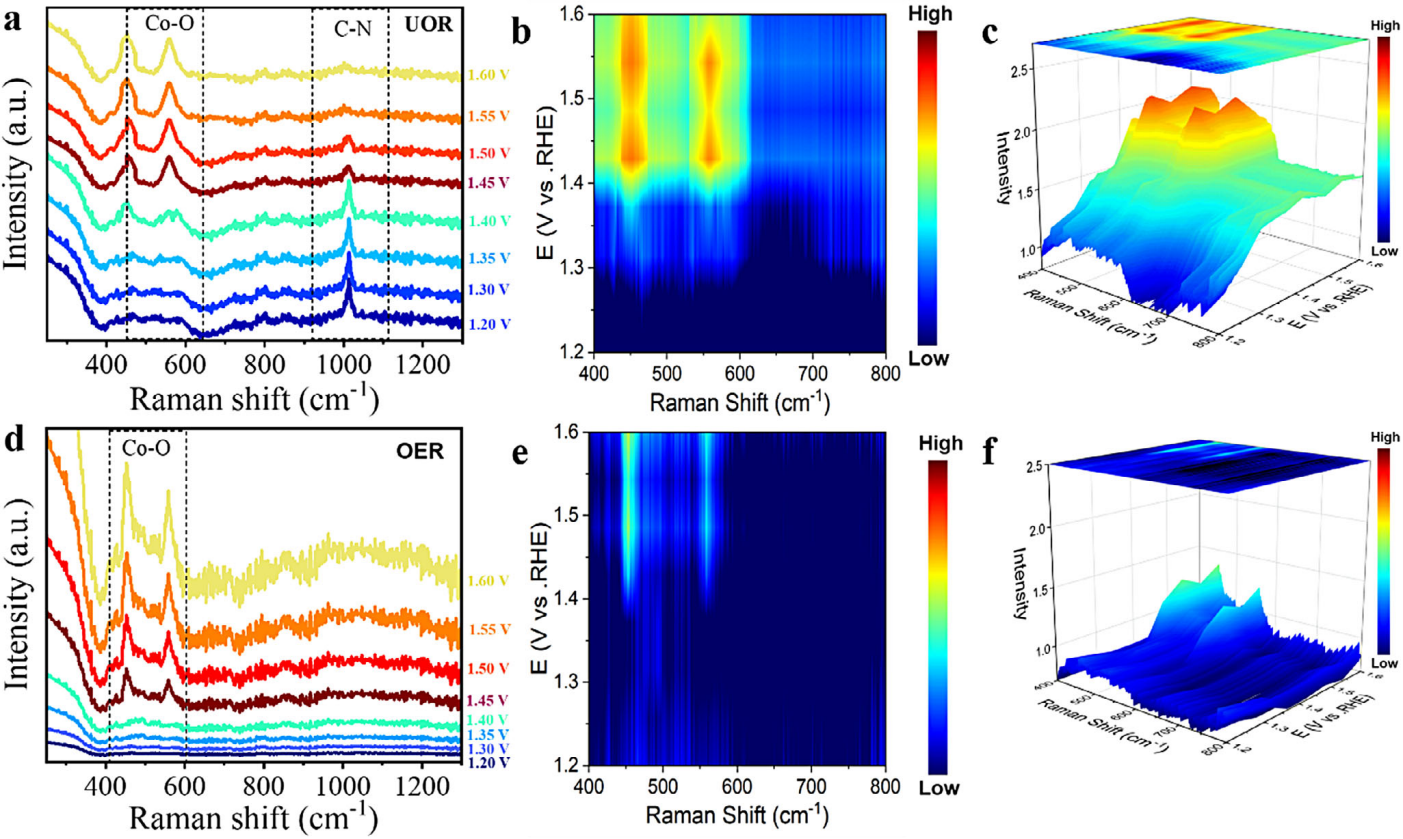
Mechanistic insight. In situ Raman spectra with corresponding contour plots and in situ FTIR spectra of urea on g‐C_3_N_4_/CoMoS_2_ collected at different potentials during UOR (a–c) and OER (d–f).

The electrochemical reaction process and the catalytic mechanism of UOR and OER were investigated using in situ Raman spectroscopy. Figure 5a–c shows the in situ Raman spectra of g‐C_3_N_4_/CoMoS_2_ in 1 m KOH solution with 0.33 m of urea, recorded at the potential range of 1.20–1.60 V (vs. RHE). Under the UOR, a pair of broad signals was observed between 400–580 cm^−1^ at a potential less than 1.40 V. These broad signal peaks split into strong doublet peaks at 453 and 565 cm^−1^ when the potential was increased beyond 1.35 V, which can be attributed to bending and stretching vibrations from Co─O, respectively, due to the formation of CoOOH [61]. Additionally, a single peak observed around 1000 cm^−1^ corresponds to the C─N stretching of urea, and this C─N stretching band continues to decrease in intensity with an increase in potential, suggesting amide bond oxidation in urea. The electrochemical formation of CoOOH and the rapid decrease in intensity of the C─N stretching band indicate the occurrence of electrochemical oxidation of Co species, as shown in Equation (2) and chemical step reaction of urea resulting in the cleavage of the C─N bond as depicted in Equation (3).

(2) $$\begin{array}{ccc} & & 6\mathrm{Co}{\left(\mathrm{OH}\right)}_{2}\left(s\right)+6OH^{-}\;\\ & & ↔6\mathrm{CoOOH}\;\left(s\right)+6H_{2}O\left(l\right)+6e^{-} \end{array}$$

(3) $$\begin{array}{ccc} & & \mathrm{CO}{\left(NH_{2}\right)}_{2}\;\left(\mathrm{aq}\right)+6\mathrm{CoOOH}\;\left(s\right)+H_{2}O\;\left(l\right)\;\\ & & \to \;N_{2}\;\left(g\right)+CO_{2}\;\left(g\right)+6\mathrm{Co}{\left(\mathrm{OH}\right)}_{2}\;\left(s\right) \end{array}$$

Under the condition of OER (Figure 5d–f), the typical characteristic peak of C─N stretch does not appear as expected, and the Raman peak arising from Co─O does not appear until the potential reaches 1.45 V, which indicates that UOR occurs at a lower potential, hence confirming more favourable than the OER under these conditions.

### 2.5 DFT Calculations Predict the Mechanism of the UOR

**FIGURE 6:**
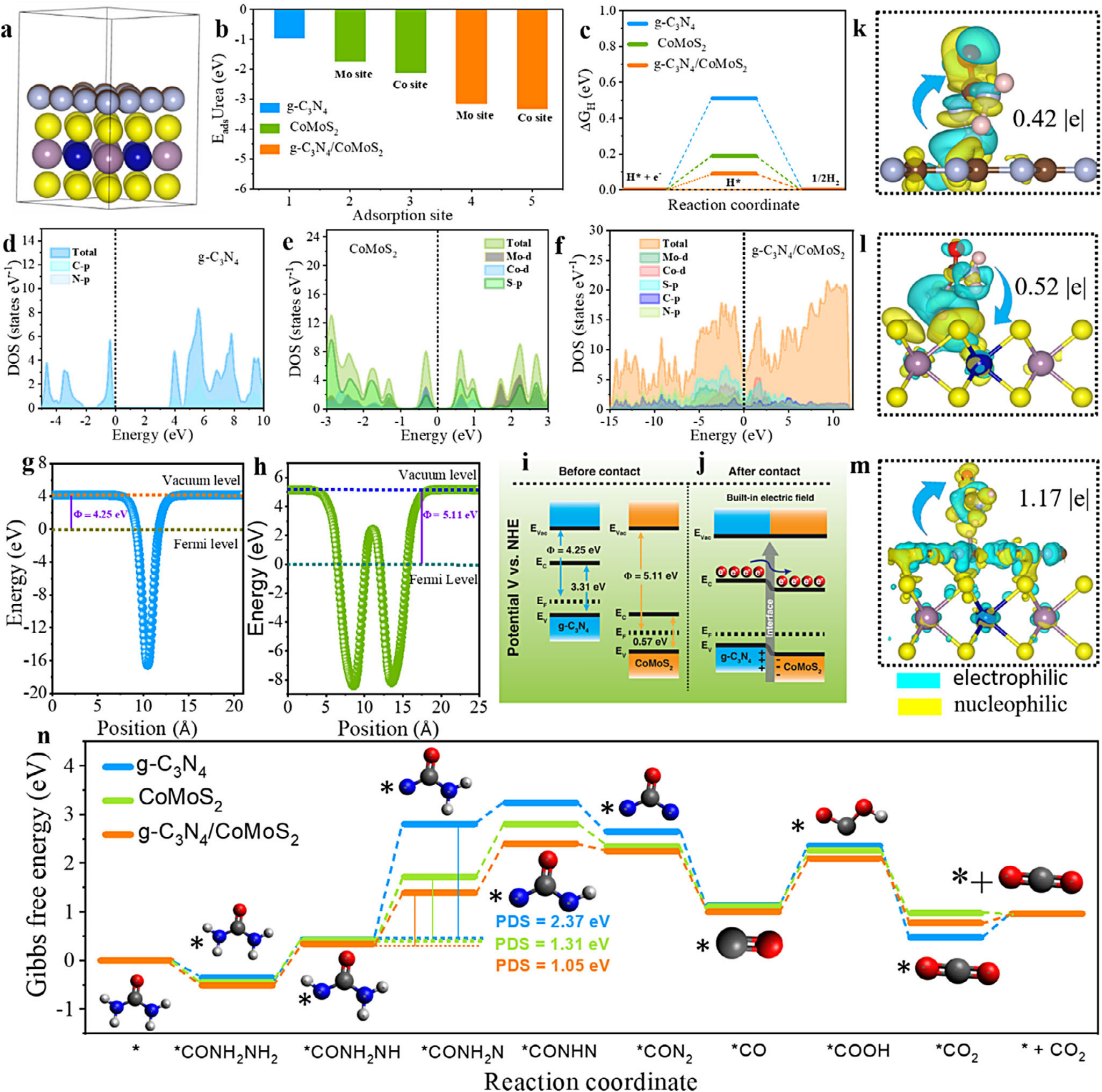
DFT Insights into the Catalytic Mechanism. (a) Optimized structural model of the g‐C_3_N_4_/CoMoS_2_ heterojunction. (b) Comparison of the adsorption energies of urea on the metal sites within the heterojunction vs. those of its individual components. (c) The H‐adsorption free energies of the g‐C_3_N_4_/CoMoS_2_ heterojunction and its individual components. DOS of (d) g‐C_3_N_4_, (e) CoMoS_2_, and (f) g‐C_3_N_4_/CoMoS_2_ heterojunction, with the Fermi level set as the reference (0 eV). The calculated 𝚽 of (g) g‐C_3_N_4_ and (h) CoMoS_2_. Schematic illustrations of the band structures of g‐C_3_N_4_ and CoMoS_2_ (i) before and (j) after interfacial contact. Charge density differences and Bader charge analysis of adsorbed urea on (k) g‐C_3_N_4_, (l) CoMoS_2_, and (m) g‐C_3_N_4_/CoMoS_2_ heterojunction. (n) Theoretical free‐energy profiles for the stepwise transformation of CO(NH_2_)_2_ into N_2_ and CO_2_ over g‐C_3_N_4_, CoMoS_2_, and the g‐C_3_N_4_/CoMoS_2_ heterojunction.

To elucidate the mechanistic origin of the distinct UOR and HER pathways on the g‐C_3_N_4_/CoMoS_2_ heterojunction, density functional theory (DFT) calculations were conducted. As a reference, structural models of pristine g‐C_3_N_4_ and CoMoS_2_ were first established (Figure S17), while the optimized atomic configuration of the g‐C_3_N_4_/CoMoS_2_ heterojunction is presented in Figure 6a. Activation of adsorbed urea at catalytic sites is a prerequisite for initiating the UOR [62]. To elucidate its kinetic implications, urea adsorption was systematically evaluated on g‐C_3_N_4_, CoMoS_2_, and the corresponding heterojunction interface. To elucidate the catalytic active site, urea was adsorbed on the basal plane of g‐C_3_N_4_ as well as the surface‐exposed Co and Mo, and the adsorption energy was systematically calculated. Representative structural configurations of urea adsorption at Co sites of CoMoS_2_ and g‐C_3_N_4_/CoMoS_2_ are illustrated in Figure S18, and the corresponding adsorption energies at various active sites are summarized in Figure 6b. Urea binds weakly to g‐C_3_N_4_ (−0.09 eV), but adsorption is substantially stronger at the Mo (−1.49 eV) and Co (−2.87 eV) sites of CoMoS_2_. Integration of g‐C_3_N_4_ with CoMoS_2_ to form the g‐C_3_N_4_/CoMoS_2_ heterojunction yields the strongest urea adsorption, as evidenced by its markedly more negative adsorption energy (−2.80 eV), underscoring the enhanced surface affinity imparted by the heterointerface. The calculated urea adsorption energy is significantly lower than that of OH^−^ (1.24 eV) on the Co site of the heterojunction surface (Figure S19), indicating preferential urea adsorption and promoting UOR over OER, in agreement with the electrochemical observations. This thermodynamic preference reflects strengthened urea‐surface interactions, facilitating the initial steps of UOR under alkaline conditions [22, 63]. Importantly, the Gibbs free energy of hydrogen adsorption (*Δ*G_H*_), key descriptor of HER activity, approaches the optimal value (≈ 0 eV) for efficient catalysts. The *Δ*G_H*_ for g‐C_3_N_4_/CoMoS_2_ (0.09 eV) is substantially lower than that of g‐C_3_N_4_ (0.51 eV) and CoMoS_2_ (0.19 eV) (Figure 6c and Figure S20), highlighting its superior hydrogen adsorption [64]. Indeed, the lower *Δ*G_H*_ for g‐C_3_N_4_/CoMoS_2_ corresponds to the experimental lower overpotential for HER. This enhancement arises from strong interfacial electronic coupling and optimized charge redistribution across the heterointerface, which collectively fine‐tune the local electronic structure and facilitate proton reduction.

To assess the conductivity of the catalysts, electronic density of states (DOS) analyses were performed (Figure 6d–f). According to the DOS analysis, g‐C_3_N_4_ and CoMoS_2_ retain their distinctive semiconducting properties, with band gaps of approximately 3.31 and 0.57 eV, respectively. In contrast, the g‐C_3_N_4_/CoMoS_2_ heterojunction exhibits a markedly increased DOS near the Fermi level, indicative of enhanced electrical conductivity and accelerated charge‐transfer kinetics. Such electronic synergy strengthens the coupling between the catalyst surface and reaction intermediates during UOR [65].

To probe the interfacial charge redistribution, the 𝚽 of g‐C_3_N_4_ and CoMoS_2_ were determined to be 4.25 and 5.11 eV, respectively (Figure 6g,h), which is close to the experimentally observed 𝚽 of 3.96 and 4.15 eV for g‐C_3_N_4_ and CoMoS_2,_ respectively. The difference in 𝚽 drives spontaneous electron transfer from g‐C_3_N_4_ to CoMoS_2_ until Fermi‐level equilibrium is reached [63], as schematically illustrated in Figure 6i,j and Figure S21. This transfer results in electron depletion in g‐C_3_N_4_ and accumulation in CoMoS_2_, rendering the latter electron‐rich. The resulting band alignment and built‐in electric field across the g‐C_3_N_4_/CoMoS_2_ interface promote charge redistribution, where electron‐enriched Co sites act as favorable adsorption centers for urea intermediates, thereby enhancing catalytic activity. To clarify the correlation between functional‐group adsorption and catalytic performance, charge‐density‐difference and Bader‐charge analyses were conducted for urea adsorption on g‐C_3_N_4_, CoMoS_2_, and the g‐C_3_N_4_/CoMoS_2_ heterojunction (Figure 6k–m). Bader analysis shows that urea acquires a more negative charge on g‐C_3_N_4_/CoMoS_2_ (−1.17 e) than on pristine g‐C_3_N_4_ (−0.42 e) and CoMoS_2_ (−0.52 e), indicating that the heterojunction enhances electron density on the adsorbed intermediate, which may facilitate stronger urea binding and lower the UOR overpotential, this observation corresponds to the experimental lower overpotential for UOR following heterojunction formation. To elucidate the complete reaction energetics, Gibbs free energy profiles were calculated for each catalytic system. As illustrated in Figure S22, the UOR proceeds through successive dehydrogenation and oxidation steps (^*^CONH_2_NH_2_ → ^*^CONH_2_NH → ^*^CONH_2_N → ^*^CONHN →^*^ CON_2_ →^*^CO →^*^ COOH → ^*^ CO_2_ → ^*^ + CO_2_) [66], consistent with previously established pathways. The corresponding free‐energy diagrams for g‐C_3_N_4_, CoMoS_2_, and g‐C_3_N_4_/CoMoS_2_ (Figure 6n and Figures S23 and S24) reveal that the second dehydrogenation step (^*^CONH_2_NH → ^*^CONH_2_N) serves as the potential‐determining step (PDS) for all catalysts [67]. Importantly, the g‐C_3_N_4_/CoMoS_2_ heterojunction exhibits a significantly reduced thermodynamic barrier of 1.05 eV compared with g‐C_3_N_4_ (2.37 eV) and CoMoS_2_ (1.31 eV), demonstrating that the heterojunction substantially lowers the reaction energy barrier. Collectively, these theoretical results confirm that heterojunction formation synergistically enhances both the kinetic and thermodynamic aspects of the UOR, consistent with the superior experimental performance of the g‐C_3_N_4_/CoMoS_2_ electrocatalyst.

### 2.6 Performance of UOR‐Assisted HER Bifunctional Electrodes

**FIGURE 7:**
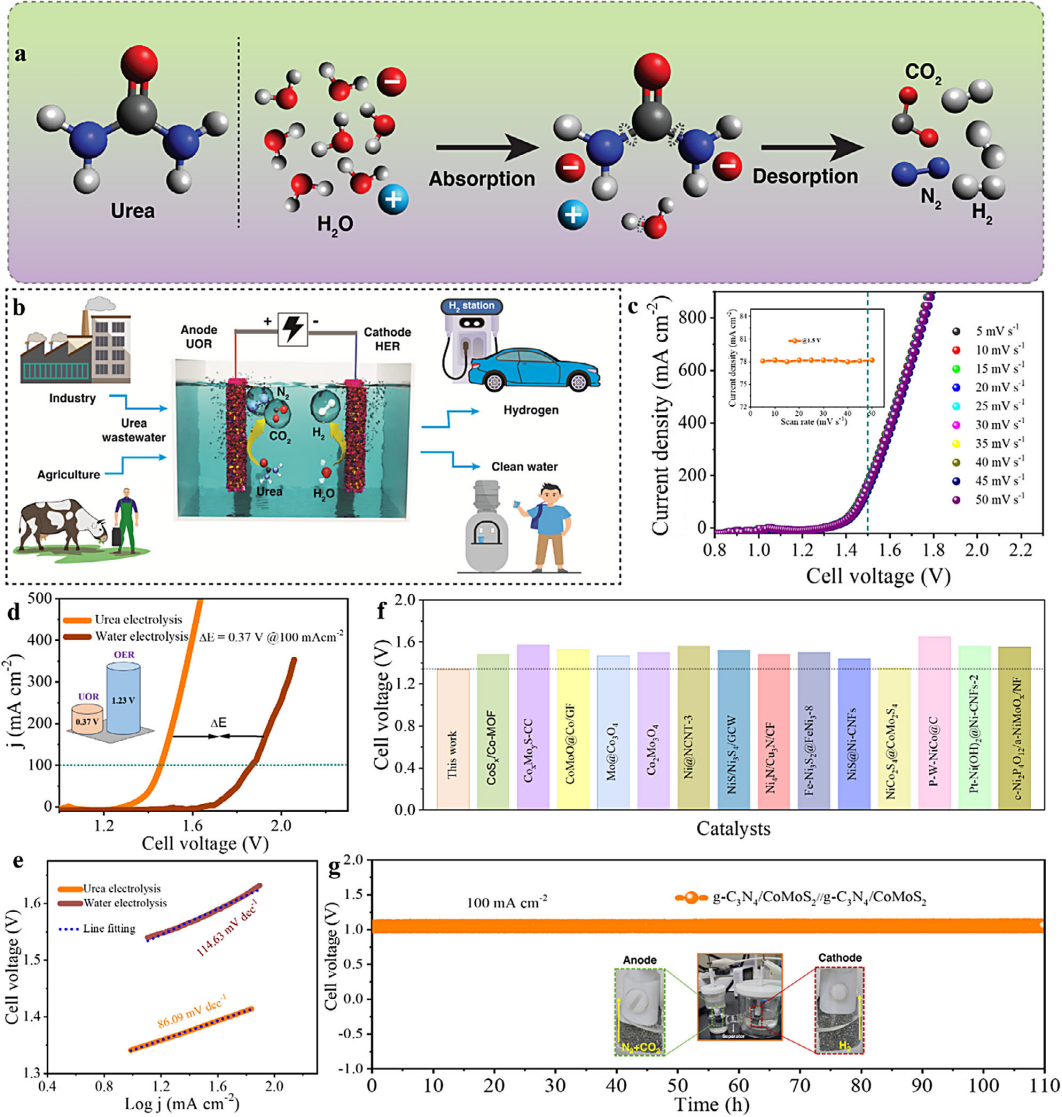
Electrocatalytic activity and durability of the UOR//HER system in 1.0 m KOH with 0.33 m urea. (a) Schematic representation of H_2_ production via urea‐assisted water splitting. (b) Schematic illustration of the overall urea electrolysis process. (c) LSV curves of g‐C_3_N_4_/CoMoS_2_ at different scan rates (inset shows the corresponding current densities at 1.50 V with different scan rates). (d) LSV curves of g‐C_3_N_4_/CoMoS_2_ measured in 1.0 m KOH with and without 0.33 M urea. (e) Tafel slopes corresponding to the two LSV curves of the UOR||HER system in 1.0 m KOH with and without 0.33 m urea. (f) Cell voltages at 10 mA cm^−2^ for g‐C_3_N_4_/CoMoS_2_ as both anode and cathode were compared with those of other reported transition‐metal‐based bifunctional electrocatalysts. (g) Long‐term electrochemical stability of g‐C_3_N_4_/CoMoS_2_ as both anode and cathode.

As schematically illustrated in Figure 7a, urea oxidation provides a thermodynamically and kinetically favorable anodic pathway, thereby substantially enhancing overall electrolysis efficiency. Accordingly, a two‐electrode urea‐assisted cell was constructed using g‐C_3_N_4_/CoMoS_2_ as both the anode and cathode in 1 m KOH containing 0.33 m urea (Figure 7b). The electrochemical behavior of g‐C_3_N_4_/CoMoS_2_ was first examined in this electrolyte configuration, with LSV curves recorded at scan rates from 5 to 50 mV s^−1^ (Figure 7c). The UOR activity remains essentially invariant across the entire scan‐rate range, spanning both low‐ and high‐current‐density regimes, indicative of rapid charge‐transfer kinetics and negligible capacitive contributions when the catalyst operates bifunctionally. For comparison, a conventional water electrolysis setup using g‐C_3_N_4_/CoMoS_2_//g‐C_3_N_4_/CoMoS_2_ in 1 m KOH was also examined. As shown in Figure 7d, the urea‐assisted cell delivers a current density of 10 mA cm^−2^ at a cell voltage of only 1.34 V, markedly lower than the 1.70 V required for standard water electrolysis. Furthermore, the g‐C_3_N_4_/CoMoS_2_//g‐C_3_N_4_/CoMoS_2_ configuration achieves current densities of 10, 30, and 50 mA cm^−2^ at remarkably low cell voltages of 1.34, 1.38, and 1.41 V, respectively, significantly below the corresponding 1.70, 1.76, and 1.80 V required under OER conditions (Figure S25). Tafel slope analysis reveals distinct kinetics, with the HER//OER pair showing a slope of 114.63 mV dec^−1^, notably higher than the 86.09 mV dec^−1^ for HER//UOR (Figure 7e). This highlights the kinetic benefits and enhanced hydrogen output of the HER//UOR pair, while avoiding the low‐value oxygen produced in conventional electrolysis, offering a practical route for domestic wastewater treatment. The g‐C_3_N_4_/CoMoS_2_ cell also exhibits one of the lowest reported cell voltages for overall urea splitting at 10 mA cm^−2^ (Figure 7f). Finally, the g‐C_3_N_4_/CoMoS_2_ stability under UOR//HER current was evaluated. Under continuous electrolysis at 100 mA cm^−2^ for 110 h, the catalyst exhibited negligible current fluctuations and maintained a stable potential, highlighting its exceptional long‐term durability (Figure 7g). To investigate the practical challenges posed by urine‐derived wastewater contamination, we performed electrolytic treatment of authentic human urine. The LSV profiles for electrolysis in 1.0 m KOH containing human urine (Figure S26) show a cell voltage of 1.48 V at 100 mA cm^−2^, indicating substantially enhanced performance relative to the 1.87 V required for water electrolysis.

## 3 Conclusion

This study overcomes the sluggish kinetics of the UOR through heterointerface engineering, wherein a built‐in electric field accelerates the reaction pathway. A robust g‐C_3_N_4_/CoMoS_2_ heterojunction was constructed through a hydrothermal and electrodeposition approach, coupling g‐C_3_N_4_ nanosheets with CoMoS_2_ nanorods to yield an efficient bifunctional electrocatalyst for both HER and UOR. This architecture enhances stability, expands the electrochemically active surface area, and facilitates efficient charge transfer. The interfacial energy disparity between g‐C_3_N_4_ and CoMoS_2_ drives spontaneous electron transfer from g‐C_3_N_4_ to CoMoS_2_, inducing a built‐in electric field that creates electrophilic sites on g‐C_3_N_4_ and nucleophilic sites on CoMoS_2_. Consequently, the heterojunction exhibits remarkable bifunctional performance, delivering potentials of −80 mV vs. RHE for HER and 1.27 V vs. RHE for UOR at 10 mA cm^−2^, with rapid kinetics and exceptional stability surpassing most state‐of‐the‐art catalysts. DFT calculations reveal that 𝚽‐driven interfacial electronic coupling directs electron transfer from g‐C_3_N_4_ to CoMoS_2_, elucidating the synergistic interactions within the heterojunction that underpin its superior electrocatalytic performance. In particular, when employed in a two‐electrode urea‐assisted cell, the heterojunction achieves a current density of 10 mA cm^−2^ at an ultralow cell voltage of 1.34 V, markedly reducing the energy demand for sustainable hydrogen production. This work demonstrates that built‐in electric fields at heterojunction interfaces modulate active‐site electronics, enabling a noble‐metal‐free catalyst for energy‐efficient urea‐assisted hydrogen production and offering a general strategy for sustainable electrocatalyst design.

## Conflicts of Interest

The authors declare no conflicts of interest.

## Supporting information




**Supporting File**: smll73842‐sup‐0001‐SuppMat.docx.

## Data Availability

The data that support the findings of this study are available on request from the corresponding author. The data are not publicly available due to privacy or ethical restrictions.
