# Supplementary material for: Molecular‐Level Engineered Approach Induces Built‐in Electric Field Modulation in G‐C3N4/CoMoS2 Heterojunction for Enhanced Hydrogen Generation via Urea Oxidation
Source: Small. 2026 May 19;22(38):e73842. doi: 10.1002/smll.73842 (PMC13351480; doi:10.1002/smll.73842)
Supplement: Supplementary file 1 — Supporting File: smll73842‐sup‐0001‐SuppMat.docx. [file SMLL-22-e73842-s001.docx]

**Supporting Information**

**Molecular-Level Engineered Approach Induces Built-in Electric Field Modulation in g-C_3_N_4_/CoMoS_2_ Heterojunction for Enhanced Hydrogen Generation via Urea Oxidation**

Boka Fikadu Banti^a^, Birhanu Bayissa Gicha^b^, Mahendra Goddati^b,c^, Hyojin Kang^d^, Indra Memdi Khoris^b^, Cheru Fekadu Molla^a^, Sohrab Asgaran^e^, Michael Giersig^f^, Njemuwa Nwaji^f*^, Jaebeom Lee^a, b,c^*

^a^ Department of Chemistry, Chungnam National University, Daejeon 34134, Republic of Korea.

^b^ Research Institute of Materials Chemistry, Chungnam National University, Daejeon 34134, South Korea.

^c^ Department of Chemical Engineering and Applied Chemistry, Chungnam National University, Daejeon 34134, Republic of Korea.

^d^ Institute for Sciences of the Universe, Chungnam National University, Daejeon 34134, Republic of Korea

^e^ Helmaco Sp. Z o.o. Company, Ostrobramska 101 / 335K, 04-041 Warszawa, Poland.

^f^ Institute of Fundamental Technological Research, Polish Academy of Sciences, Pawinskiego 5B Str. 02-106, Warsaw, Poland.

*Corresponding authors email

Nwaji Njemuwa; [nnwaji@ippt.pan.pl](mailto:nnwaji@ippt.pan.pl), Jaebeom Lee; [nanoleelab@cnu.ac.kr](mailto:nanoleelab@cnu.ac.kr)

**1 Experimental Section**

**1.1 Chemicals**

Thiourea, nickel foam (NF), cobalt (II) nitrate hexahydrate (Co(NO_3_)_2_·6H_2_O), deionized water, ethanol, urea, ammonium molybdate tetrahydrate ((NH_4_)_6_Mo_7_O_24_·4H_2_O), Pt/C, RuO_2_, and potassium hydroxide (KOH) were used as received, without any further purification.

**1.2 Synthesis of g-C_3_N_4_ nanosheets**

Graphitic carbon nitride (g-C_3_N_4_) was prepared through a straightforward thermal polymerization approach. In a typical procedure, 5 g of thiourea was loaded into an alumina crucible with a covered lid and calcined at 580 °C for 4 h in a tubular furnace under ambient atmosphere. After naturally cooling to room temperature, the resulting pale-yellow powder was collected and thoroughly ground to obtain a uniform product for subsequent applications.

**1.3 Synthesis of CoMoS_2_**

Before synthesis, NF (1.0 × 0.1 × 2.0 cm) was sequentially ultrasonicated in diluted HCl, acetone, deionized water, and anhydrous ethanol to remove surface oxides and organic residues, followed by drying overnight in a vacuum oven. In a typical procedure, Co(NO_3_)_2_·6H_2_O (0.2 g), (NH_4_)_6_Mo_7_O_24_·4H_2_O (0.1 g), and thiourea (0.05 g) were dissolved in 25 mL of deionized water under continuous stirring to form a clear precursor solution. The pretreated nickel foam was immersed in this solution and transferred to a Teflon-lined stainless-steel autoclave, maintained at 150 °C for 5 h. After natural cooling to room temperature, the obtained product was rinsed thoroughly with deionized water and ethanol, followed by vacuum drying to yield CoMoS_2_.

**1.4 Synthesis of g-C_3_N_4_/CoMoS_2_ heterojunction**

The g-C_3_N_4_/CoMoS_2_ heterojunction was fabricated through a streamlined electrodeposition protocol. The electrodeposition of g-C_3_N_4_ nanosheets onto CoMoS_2_/NF was carried out under potentiostatic conditions, with the applied electric field as the primary driving force for deposition. Under the applied potential, charged or protonated g-C_3_N_4_ species in the electrolyte migrate directionally toward the conductive electrode surface via electrophoretic transport. Upon reaching the CoMoS_2_/NF substrate, these species undergo electrostatic adsorption, followed by assembly. The high electrical conductivity of the CoMoS_2_ nanorod arrays facilitates efficient electron transfer, promoting localized nucleation and firm anchoring of g-C_3_N_4_ nanosheets. In addition, interfacial interactions, including electrostatic attraction and possible coordination between surface functional groups (e.g., –NHₓ) and exposed metal sites, further stabilize the deposited layer, yielding a uniform, strongly coupled heterostructure. Briefly, g-C_3_N_4_ was electrochemically deposited onto pre-cleaned and dried CoMoS_2_/NF using a conventional three-electrode configuration, with CoMoS_2_/NF, Pt foil, and Ag/AgCl serving as the working, counter, and reference electrodes, respectively. The deposition electrolyte consisted of 20 mg of g-C_3_N_4_ dispersed in 40 mL of distilled water containing 1.0 M KOH and stirred until a homogeneous solution was obtained. Cyclic voltammetry was then conducted between -1.2 and 0.2 V at a scan rate of 5 mV s^-1^ for five cycles to achieve conformal growth of g-C_3_N_4_. Following deposition, the electrodes were rinsed with distilled water and vacuum-dried at 60 °C for 12 h

**1.5. Materials Characterization**

The morphological and structural features of the synthesized materials were systematically investigated using advanced characterization techniques. Field-emission scanning electron microscopy (FESEM, Merlin Compact, Carl Zeiss, Germany) was employed to evaluate the surface morphology. Transmission electron microscopy (TEM) and high-resolution transmission electron microscopy (HRTEM) analyses were performed on a Tecnai G2 F30 S-TWIN (FEI, USA) and a JEM-3010 (JEOL, Japan), respectively, to examine the nanostructure and lattice fringes. Elemental composition and spatial distribution were analyzed via energy-dispersive X-ray spectroscopy (EDS) coupled with an AZtech Energy_X–MaxN system (Oxford Instruments, UK). X-ray photoelectron spectroscopy (XPS, MultiLab 2000, Thermo Scientific, USA) was conducted to probe the surface composition and chemical states of elements. X-ray diffraction (XRD) patterns were collected on a MiniFlex II diffractometer (Rigaku, Japan) using Cu Kα radiation (λ = 1.5406 Å) operated at 30 kV, with a scanning rate of 0.05° min⁻¹, to evaluate the crystallographic phase and structural integrity of the materials.

**1.6. Electrochemical Measurements**

Electrochemical characterizations, including cyclic voltammetry (CV), linear sweep voltammetry (LSV), electrochemical impedance spectroscopy (EIS), and chronopotentiometry, were performed using an IviumStat electrochemical workstation (Ivium Technologies, Eindhoven, Netherlands) in a standard three-electrode configuration. Ag/AgCl electrode (3 M KCl) and a Pt wire were employed as the reference and counter electrodes, respectively. The working electrode consisted of nickel foam (NF, 1 × 2 cm^2^) modified with the synthesized catalyst via direct hydrothermal growth and electrodeposition. All electrode potentials were converted to the reversible hydrogen electrode (RHE) scale using the Nernst equation:

$E_{RHE}=E_{Ag/AgCl}+0.197V+0.0591 x pH$ (1)

The hydrogen evolution reaction (HER) was evaluated in 1.0 M KOH solution, while urea oxidation reaction (UOR) measurements were conducted in 1.0 M KOH containing 0.33 M urea (specific gravity 1.003) or 1.0 M KOH containing human urine (specific gravity 1.025) at a scan rate of 5 mV s^-1^. Before each measurement, the working electrodes were activated by repeated CV scanning until stable response profiles were obtained. EIS was performed over a frequency range of 100 kHz to 0.1 Hz using a perturbation amplitude of 5 mV. Chronopotentiometry measurements were carried out to assess catalytic stability under constant current operation. All polarization curves were corrected for solution resistance (iR compensation) using a 95% positive feedback method, unless stated otherwise. The electrochemical double-layer capacitance (C_dl_) was evaluated by CV scans at various scan rates (10 -80 mV s⁻¹) within a non-faradaic potential window in 1.0 M KOH. The slope of the linear fit of current density versus scan rate was used to determine C_dl_. The electrochemically active surface area (ECSA) was estimated according to the equation:

$ECSA=\frac{C_{dl}}{C_{s}}$ (2)

where C_dl_ is double layer capacitance, C_s_ is specific capacitance and was assumed to be 0.040 mF cm^-2^ in alkaline solution.

**1.7. Computational Methods**

Density functional theory (DFT) calculations were carried out using the Quantum ESPRESSO package to investigate the structural, electronic, and catalytic properties of g-C_3_N_4_, CoMoS_2_, and g-C_3_N_4_/CoMoS₂ heterojunction [1, 2]. The electron-ion interactions were modeled using the projector augmented wave (PAW) method, and the Kohn-Sham orbitals were expanded using a plane-wave basis set with a kinetic energy cutoff of 50 Ry, which ensured convergence of the total energy. The exchange correlation interactions were treated using the generalized gradient approximation (GGA) with the Perdew–Burke–Ernzerhof (PBE) functional. The convergence thresholds for self-consistent field (SCF) iterations and atomic forces were set to 1 × 10^-5^ eV and 0.01 eV Å^-1^, respectively. To model surface interactions, models of g-C_3_N_4_, CoMoS_2_, and g-C_3_N_4_/CoMoS_2_ interfaces were constructed based on experimental HRTEM and XRD data. A 15 Å vacuum layer was introduced along the z-axis to prevent interactions between periodic images. The Gibbs free energy for HER in alkaline conditions and UOR was determined according to the following equation:

${\Delta G}_{H^{*}}={\Delta E}_{H^{*}}+{\Delta E}_{ZPE}-T\Delta S$ **(S1)**

where ${\Delta E}_{H^{*}}$, ${\Delta E}_{ZPE}$, and $\Delta S$ reflect the binding energy of the H intermediate, the zero-point energy change, and the entropy change, respectively. In this work, we calculated the ${\Delta E}_{{urea}^{*}}$ based on the following equation:

${\Delta E}_{{urea}^{*}}=E_{\left( surf+{urea}^{*} \right)}-E_{\left( surf \right)}-E_{urea}$ **(S2)**

where $E_{\left( surf+{urea}^{*} \right)}$ represents the total system energy with adsorbed urea intermediate in each unit cell. $E_{\left( surf \right)}$ and $E_{urea}$ represents the energy of the bare surface and the urea gas molecule, respectively.


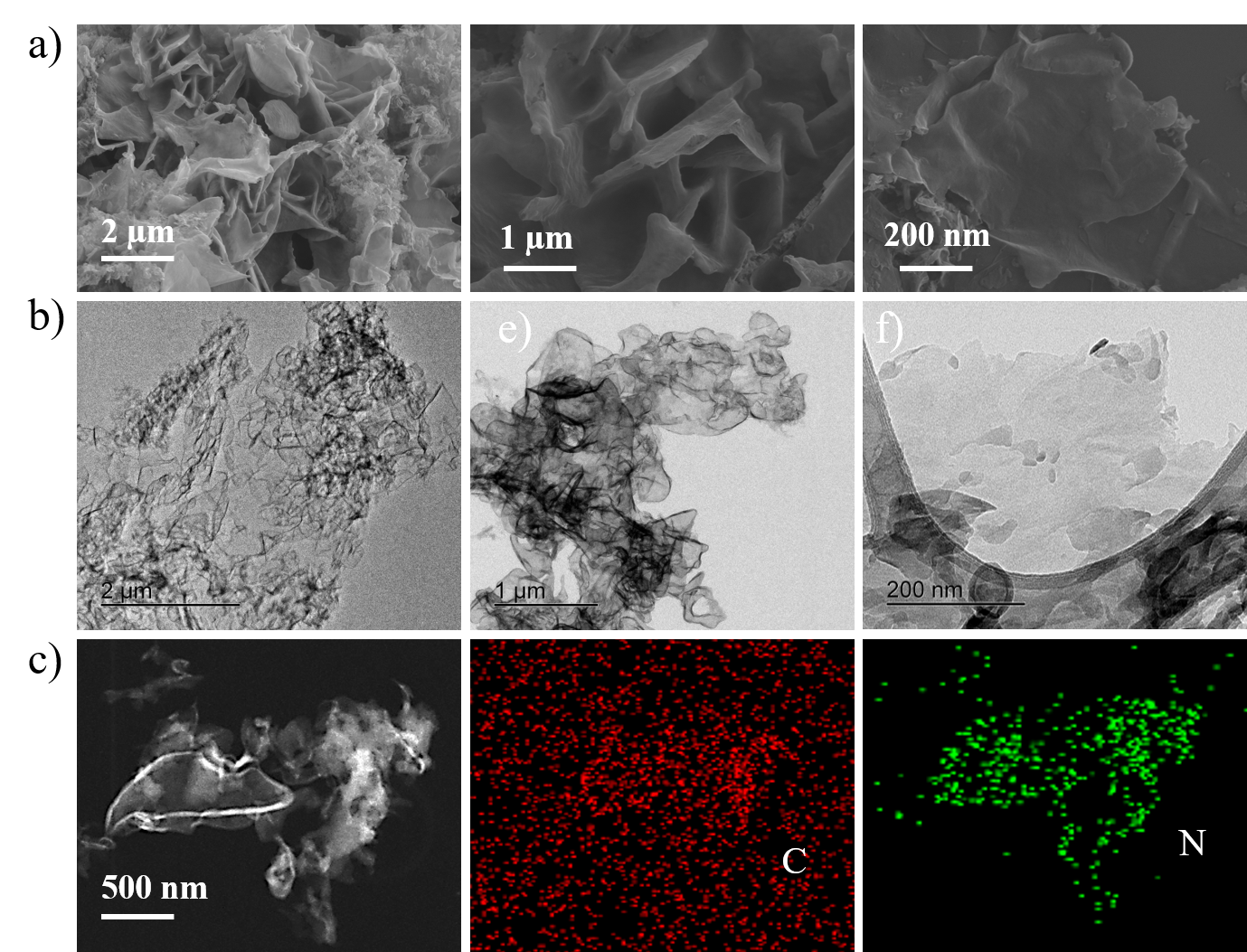


**Figure S1.** Morphological characterization of g-C_3_N_4_. a) SEM images at different magnifications, b) TEM images at different magnifications, and c) TEM-energy-dispersive X-ray spectroscopy (EDS) elemental mappings of g-C_3_N_4_.


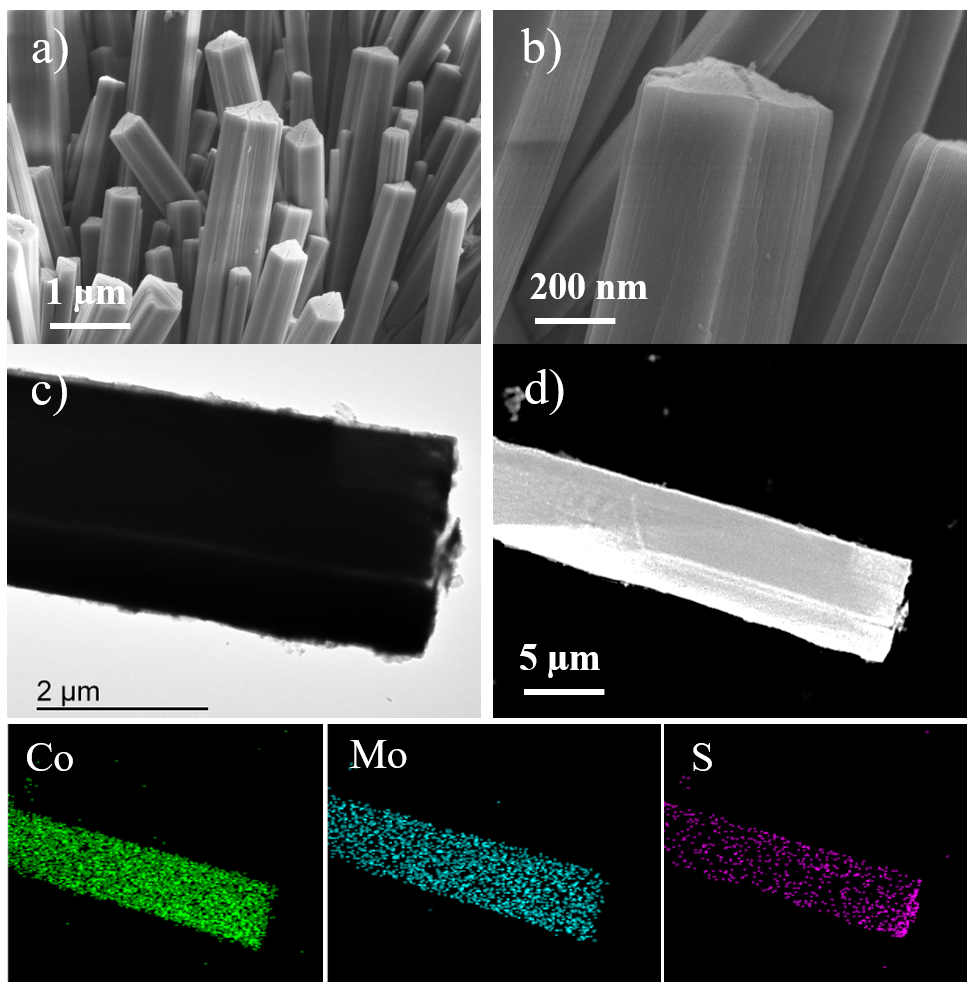


**Figure S2.** Morphological characterization of pristine CoMoS_2_. a,b) SEM images at different magnifications, c) TEM images, and d) TEM-energy-dispersive X-ray spectroscopy (EDS) elemental mappings of CoMoS_2_.


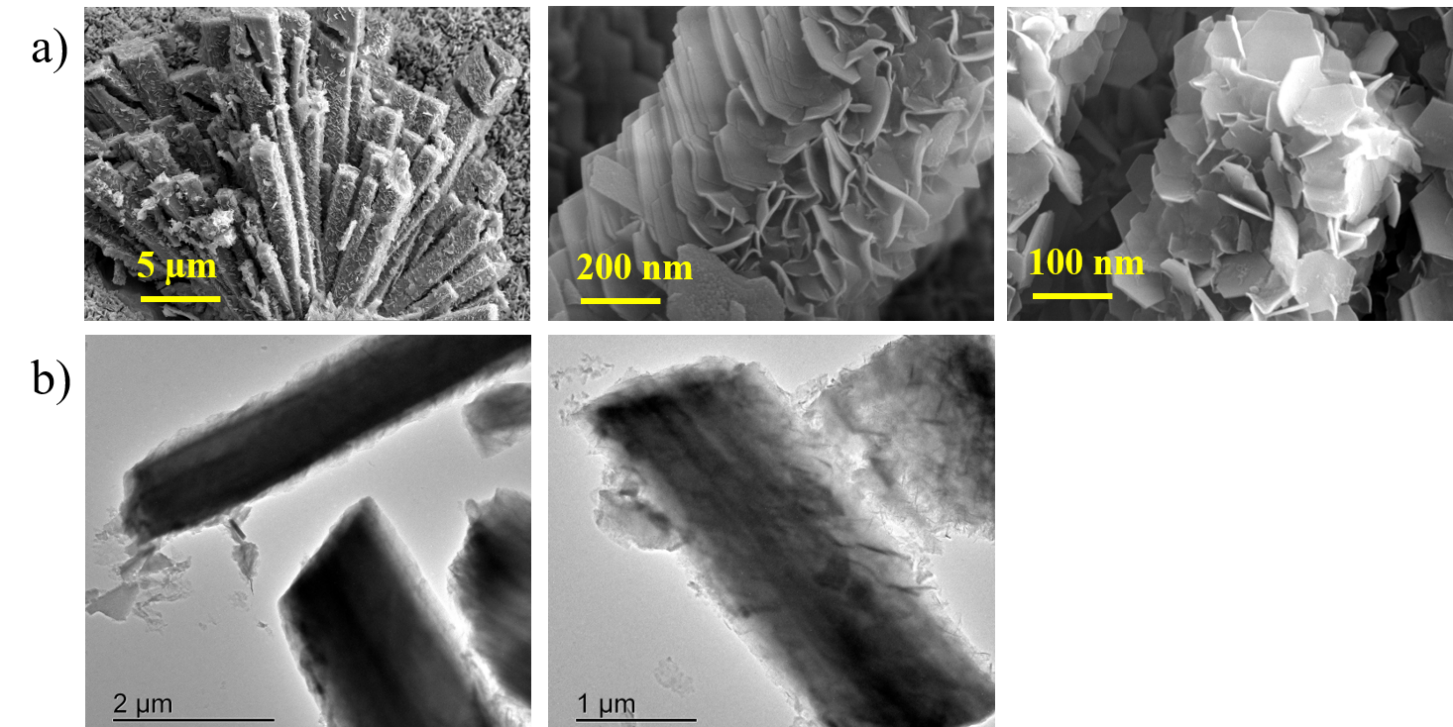


**Figure S3.** Morphological characterization of g-C_3_N_4_/CoMoS_2_ heterojunction. a) SEM images at different magnifications, b) TEM images at different magnifications of g-C_3_N_4_/CoMoS_2_.

 
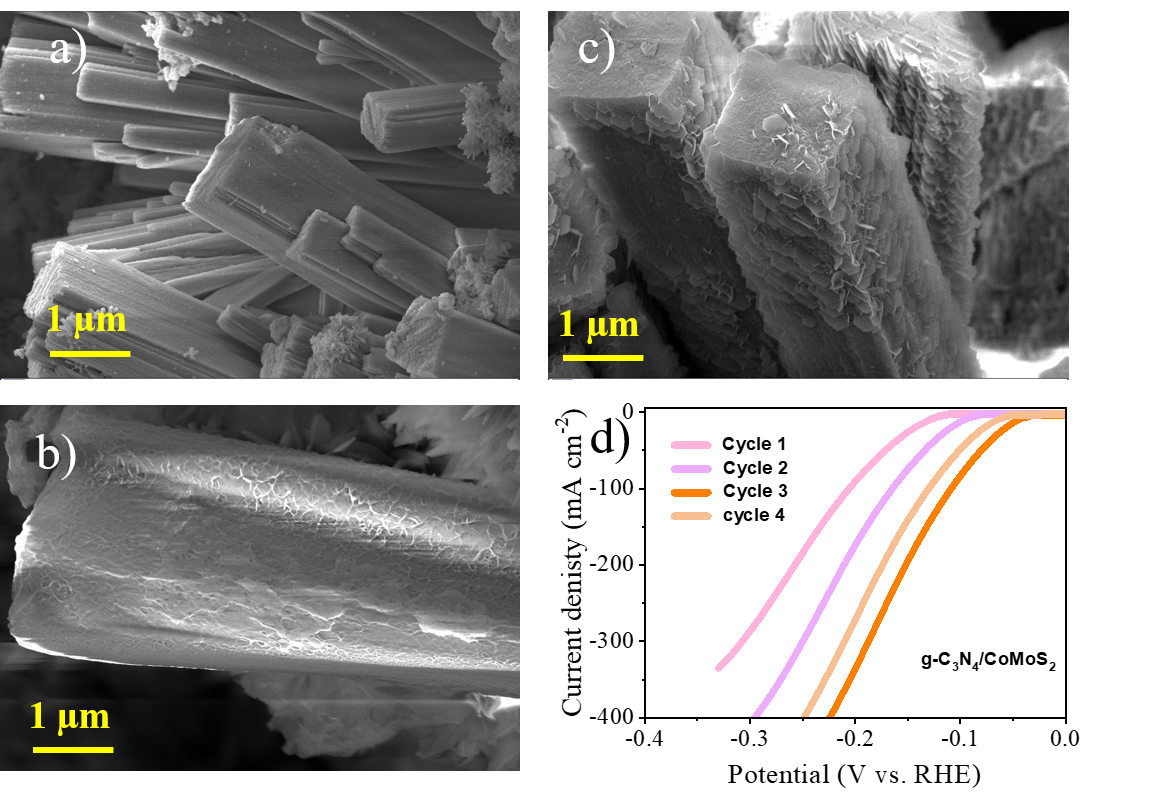


**Figure S4.** Morphological evolution and electrochemical performance of g-C_3_N_4_/CoMoS_2_ heterostructure. (a-c) SEM images of the heterostructures obtained after different electrodeposition cycles: (a) cycle 1, (b) cycle 2, (c) cycle 4, showing progressive surface coverage and structural transformation. (d) HER polarization curves of g-C_3_N_4_/CoMoS_2_ electrodes prepared under different electrodeposition conditions.


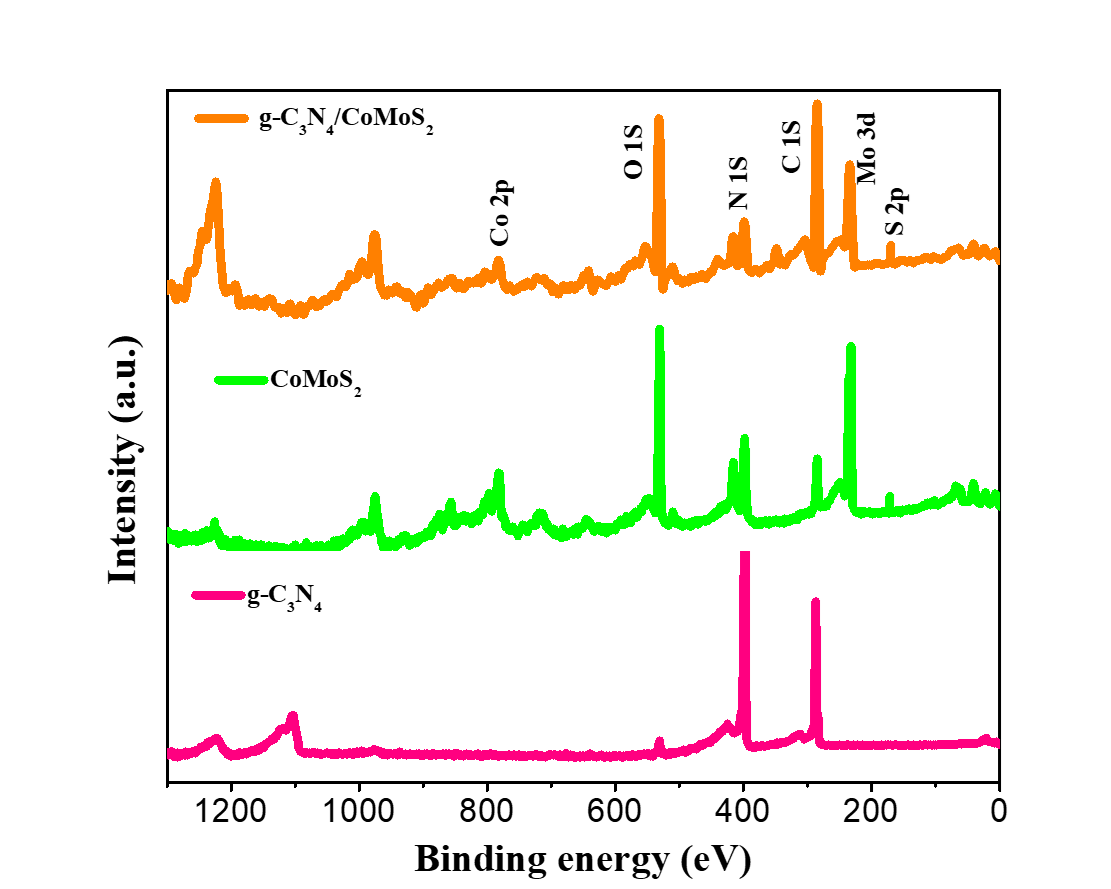
 **Figure S5**. XPS survey spectra of g-C_3_N_4_, CoMoS_2_, and g-C_3_N_4_/CoMoS_2_


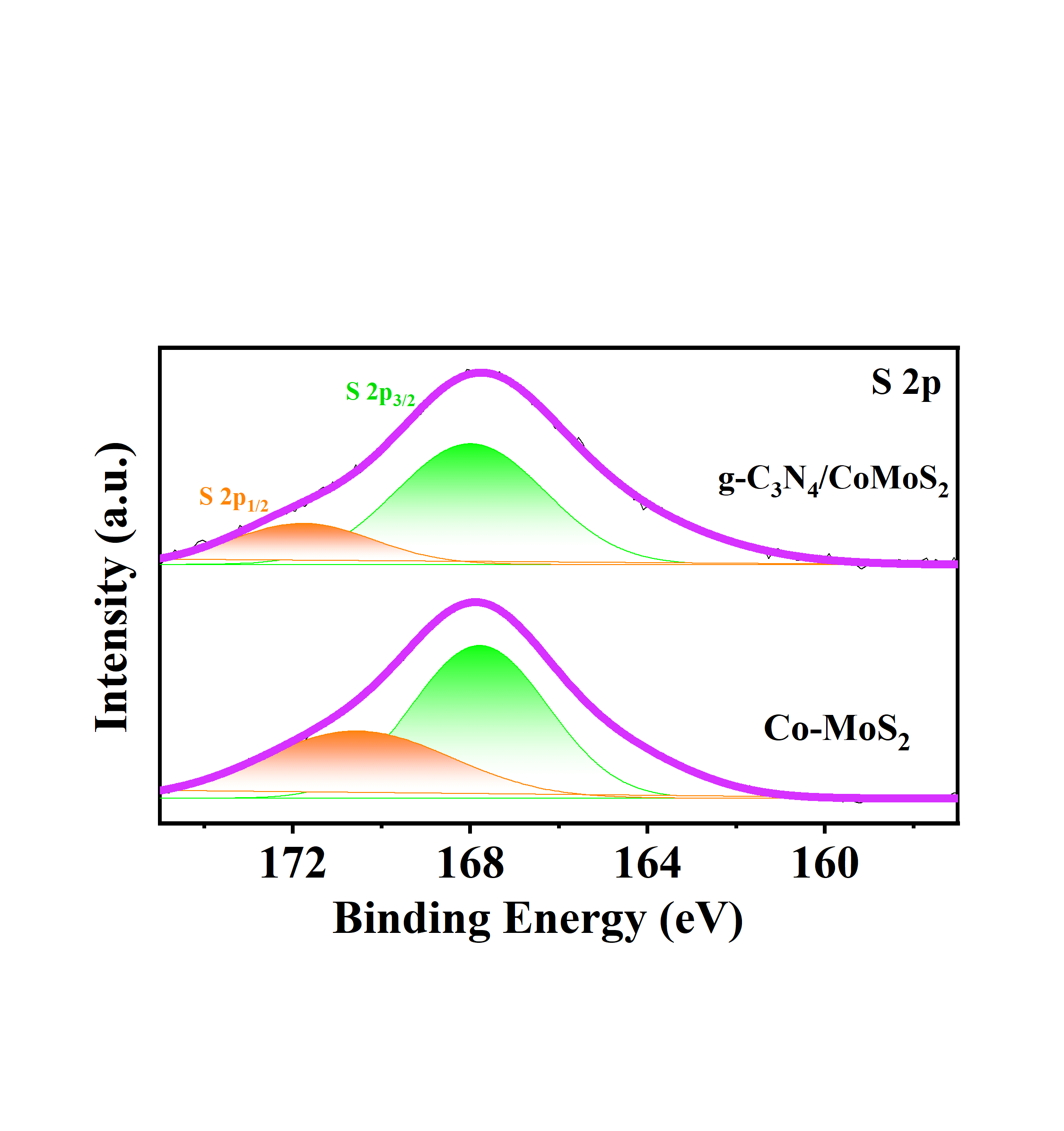


**Figure S6**. High-resolution XPS spectra of CoMoS_2_ and g-C_3_N_4_/CoMoS_2_ for S 2p.


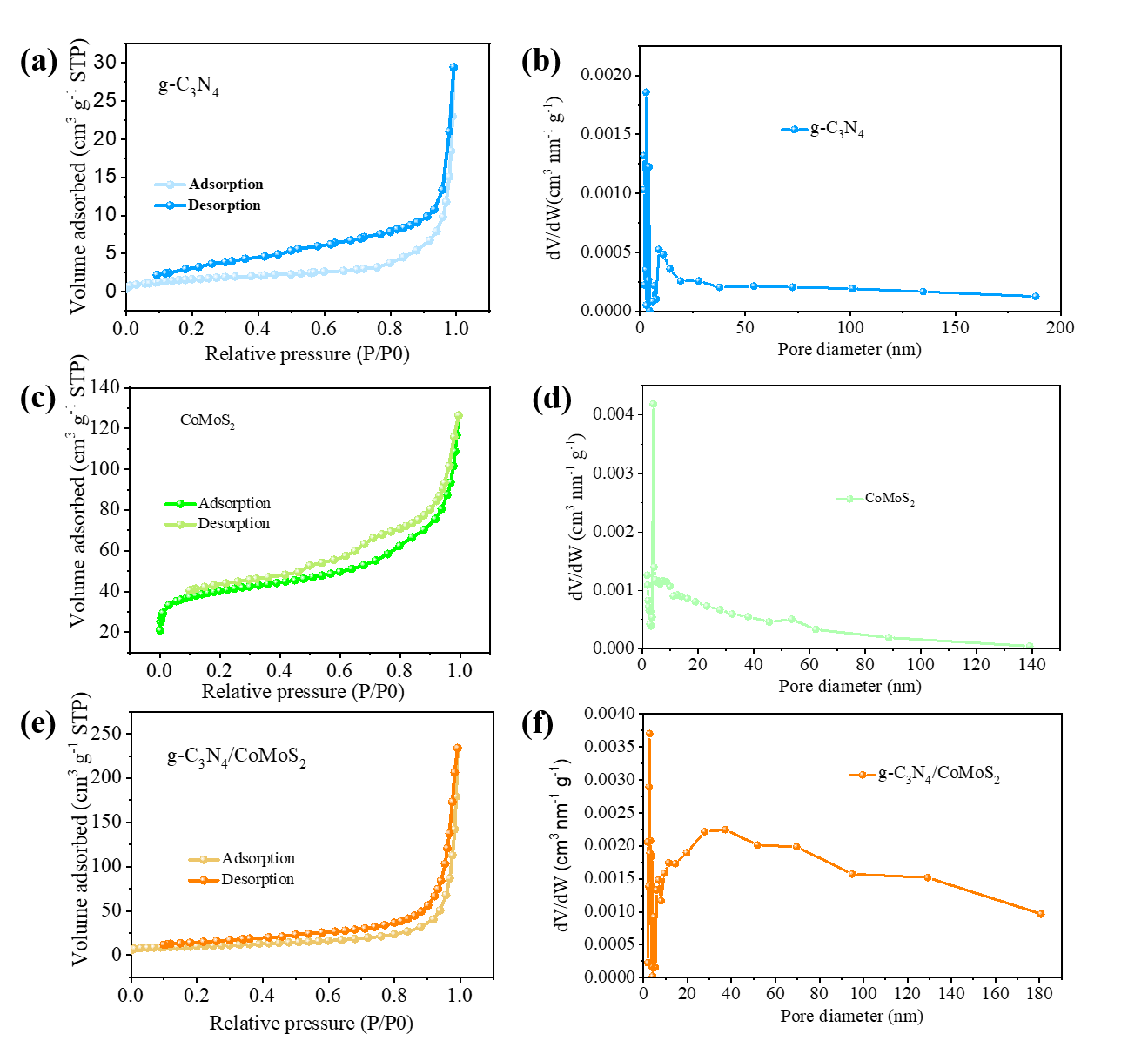


**Figure S7.** (a) N_2_ adsorption-desorption isotherms and (b) corresponding pore size distribution curves of g-C_3_N_4_. (c) N_2_ adsorption-desorption isotherms and (d) corresponding pore size distribution curves of CoMoS_2_. (e) N_2_ adsorption-desorption isotherms and (f) corresponding pore size distribution curves of g-C_3_N_4_/CoMoS_2_ heterojunction.

**Table S1.** Textural properties of the heterojunction and its individual components derived from N_2_ adsorption–desorption measurements.

| Sample | BET Surface Area (m^2^ g^-1^) | Pore Volume (cm^3^ g^-1^) |
| --- | --- | --- |
| g-C_3_N_4_ | 6.26 | 0.045 |
| CoMoS_2_ | 12.3671 | 0.085 |
| g-C_3_N_4_/CoMoS_2_ | 37.5428 | 0.134 |


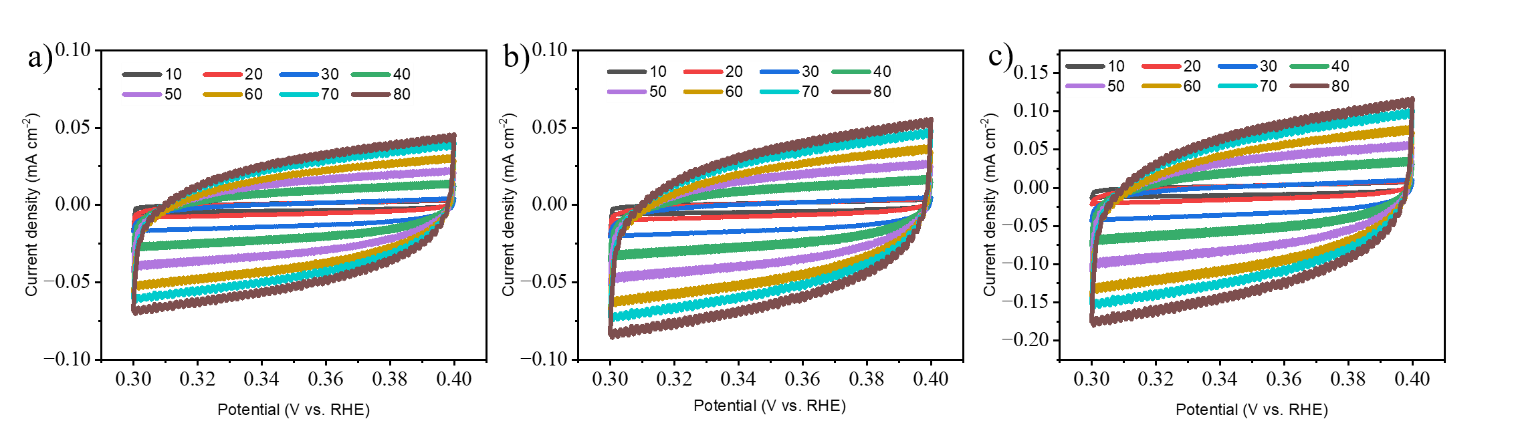


**Figures S8.** CV curves of a) g-C_3_N_4_, b) CoMoS₂, and c) g-C_3_N_4_/CoMoS_2_ recorded at various scan rates for electrochemical double-layer capacitance (C_dl_) estimation.


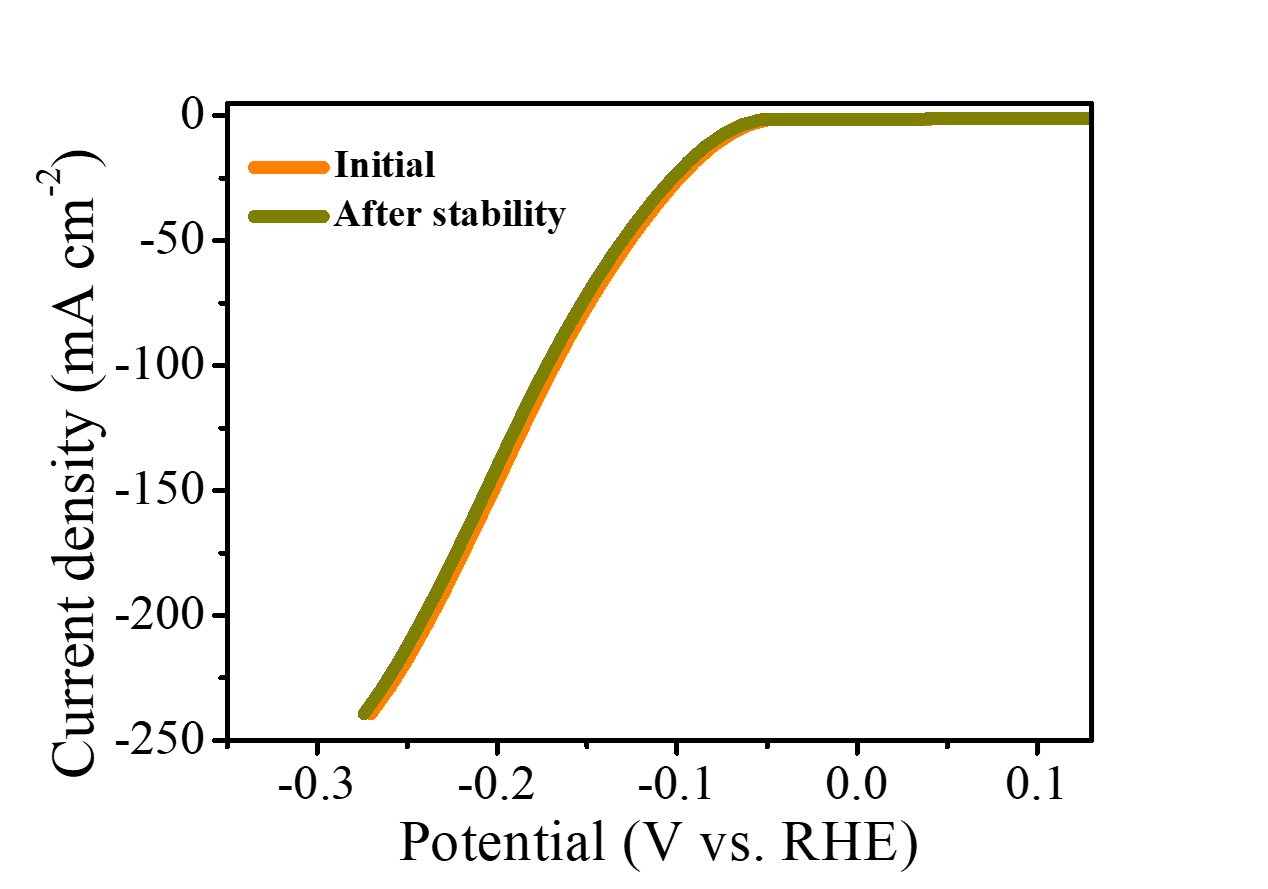


**Figure S9**. HER polarization plots of g-C_3_N_4_/CoMoS_2_ in 1 M KOH electrolyte before and after stability testing at 10 mA cm^-2^.


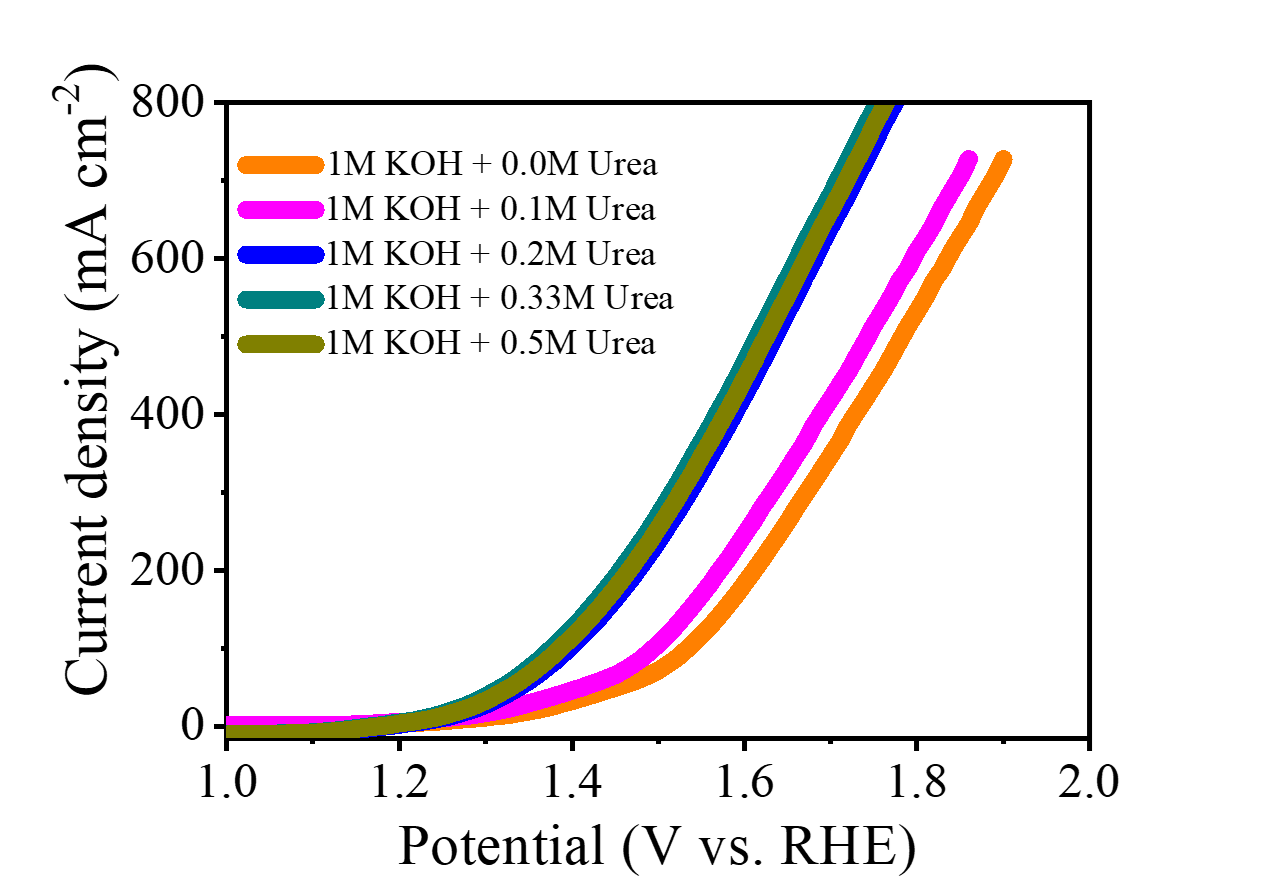


**Figure S10**. Polarization curves of g-C_3_N_4_/CoMoS_2_ at different concentrations of urea.


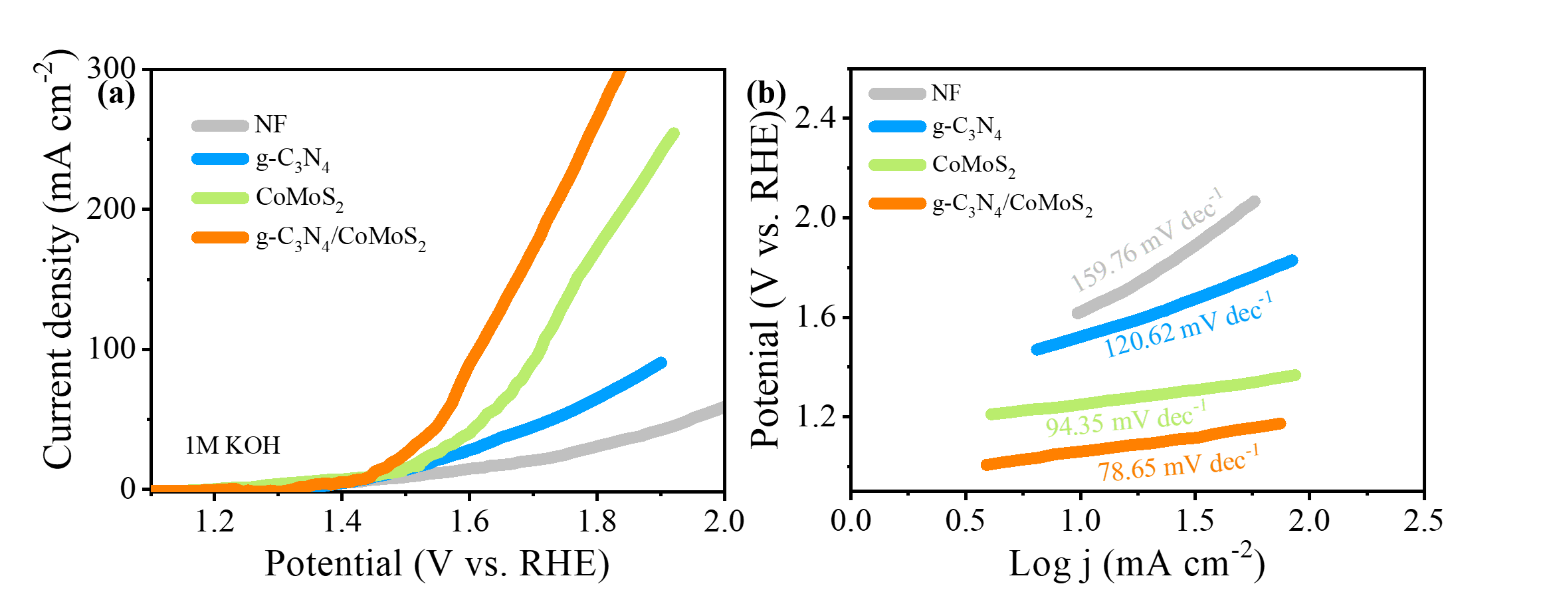


**Figure S11**. (a) LSV polarization curves of the investigated samples in 1 M KOH electrolyte without urea and (b) Corresponding Tafel slopes.


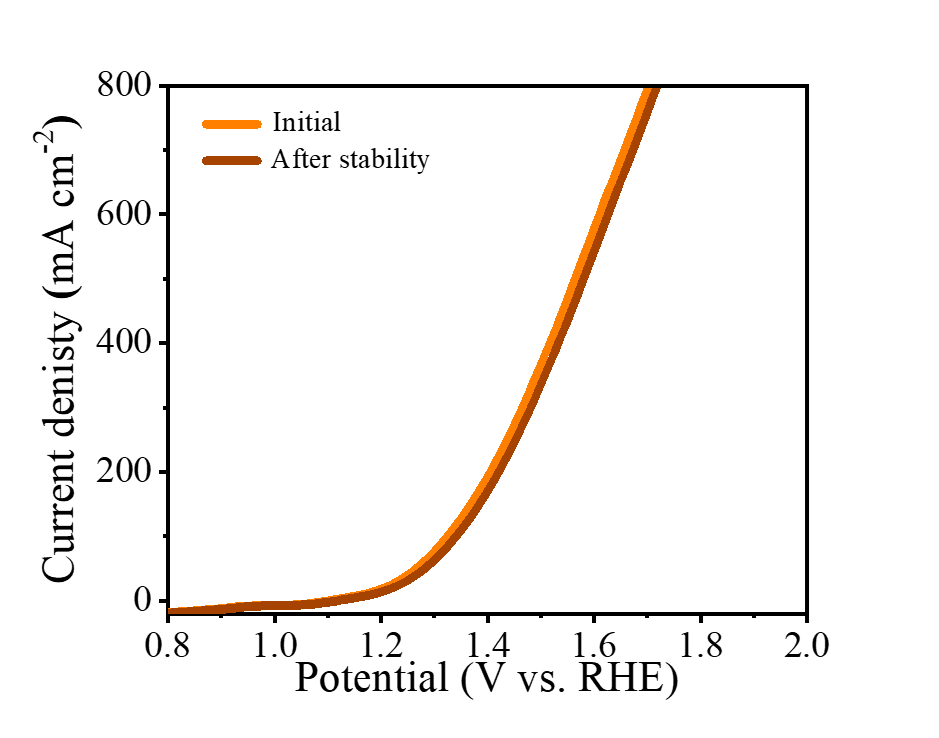


**Figure S12.** LSV curves before and after stability test of the g-C_3_N_4_/CoMoS_2_ catalyst.


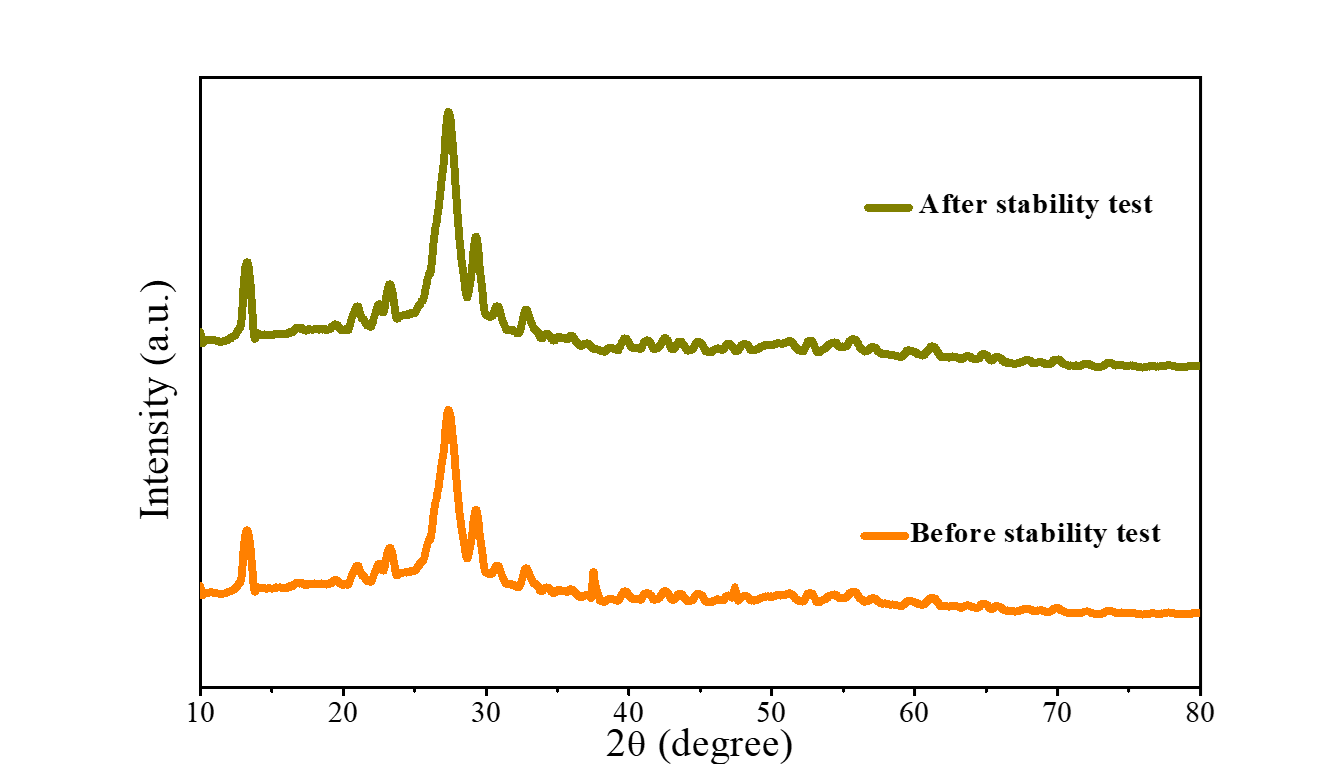


**Figure S13**. XRD patterns of the g-C_3_N_4_/CoMoS_2_ heterojunction recorded before and after 120 h of continuous UOR operation, highlighting its structural stability.


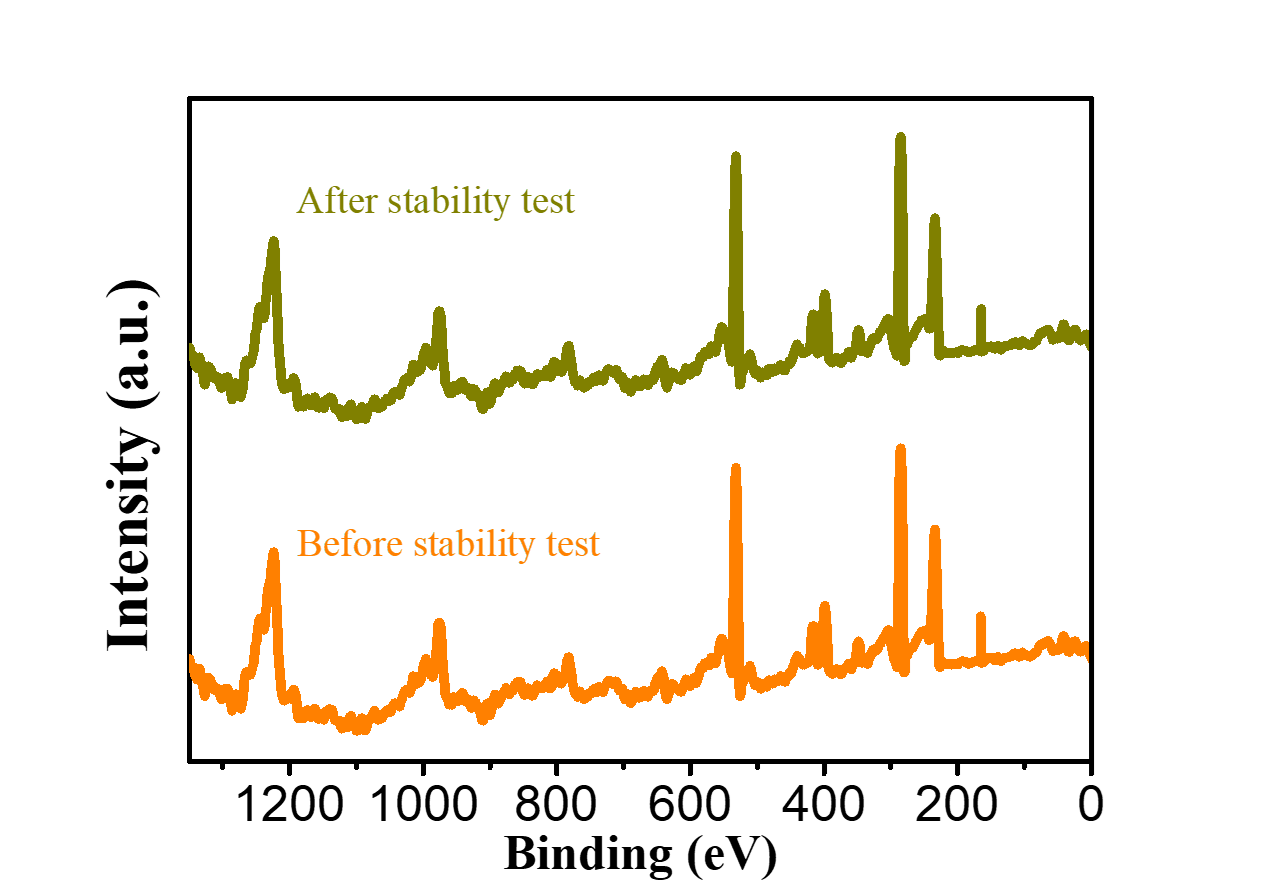


**Figure S14.** XPS survey spectra of g-C_3_N_4_/CoMoS_2_ before and after stability test.


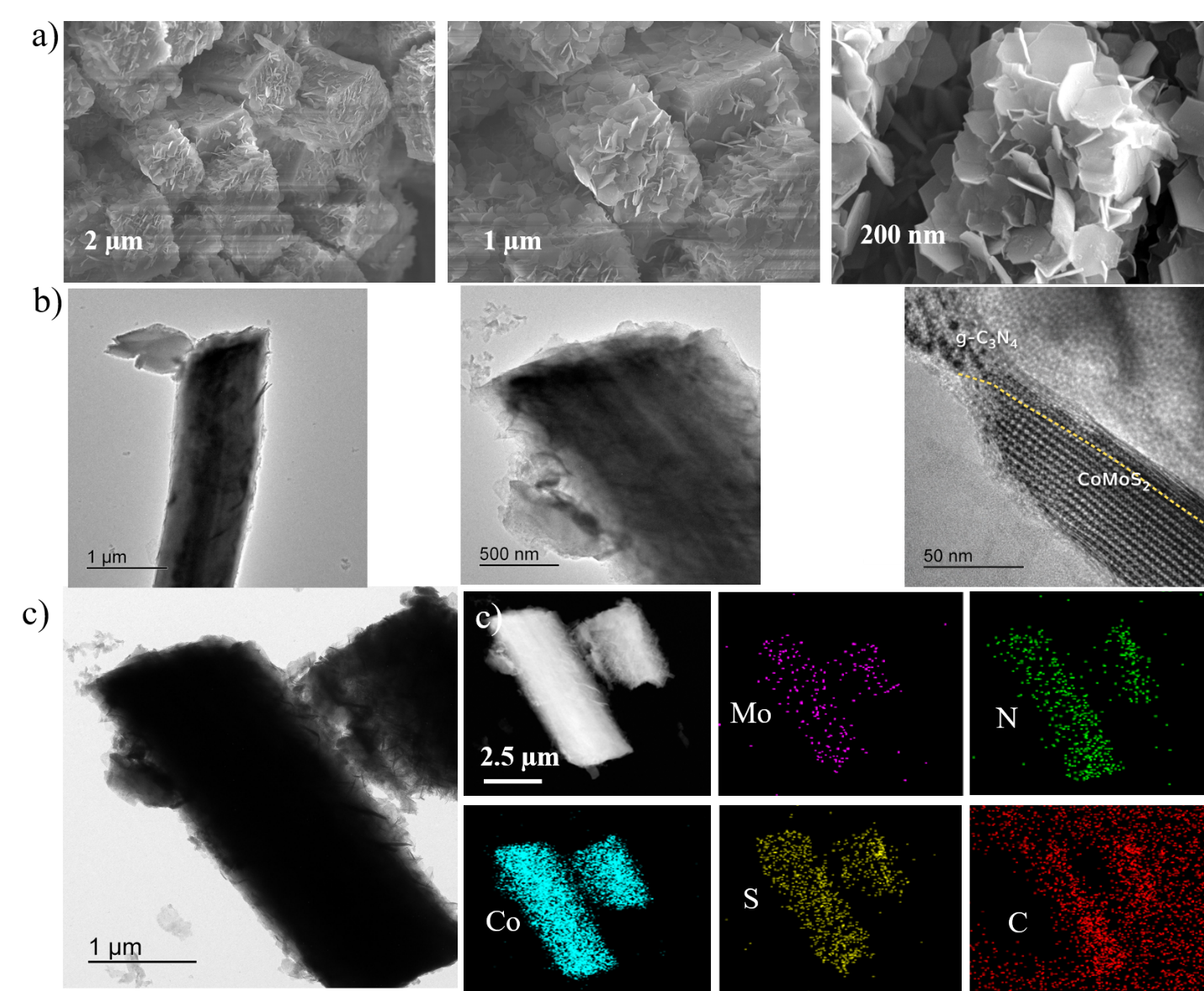


**Figure S15.** Morphological characterization of g-C_3_N_4_/CoMoS_2_ heterojunction after stability test. a) SEM images at different magnifications, b) TEM images and high-resolution TEM (HR-TEM) image of g-C_3_N_4_/CoMoS_2_, and c) TEM-energy-dispersive X-ray spectroscopy (EDS) elemental mappings of g-C_3_N_4_/CoMoS_2_ after 120 h of continuous UOR operation, highlighting its morphological stability.


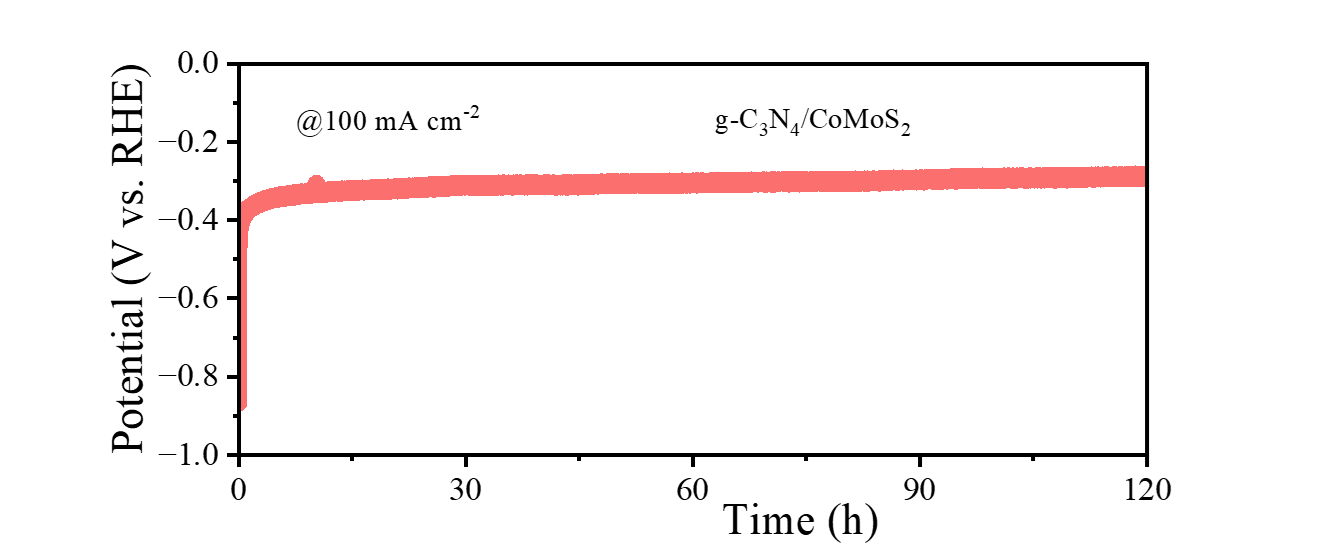
**Figure S16**. Chronopotentiometric stability test of g-C_3_N_4_/CoMoS_2_ at 100 mA cm^-2^ in the 1.0 M KOH + urine.


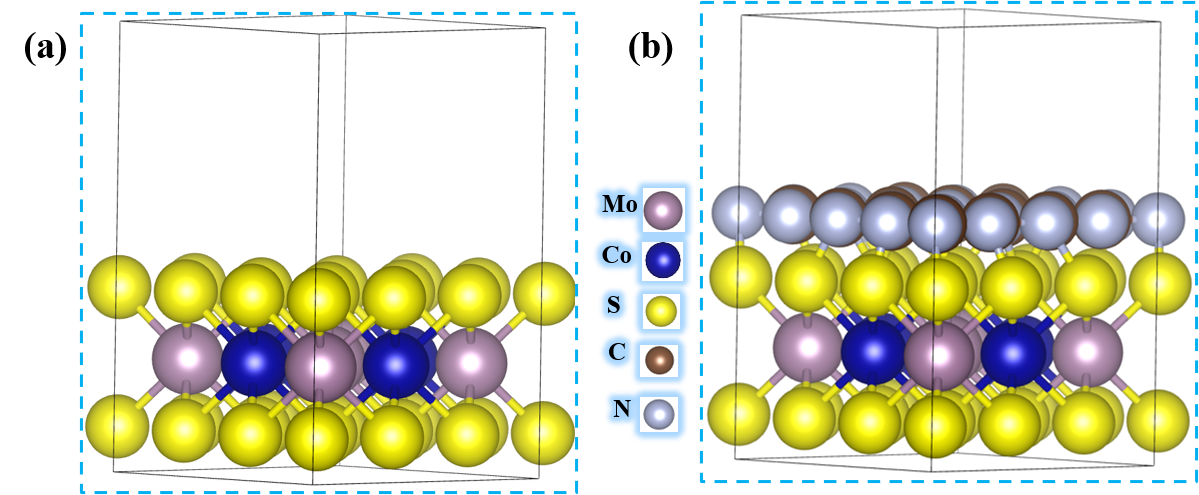


**Figure S17.** Schematic presentation of the atomic structures of a) CoMoS_2_ and b) g-C_3_N_4_/CoMoS_2_ heterojunction


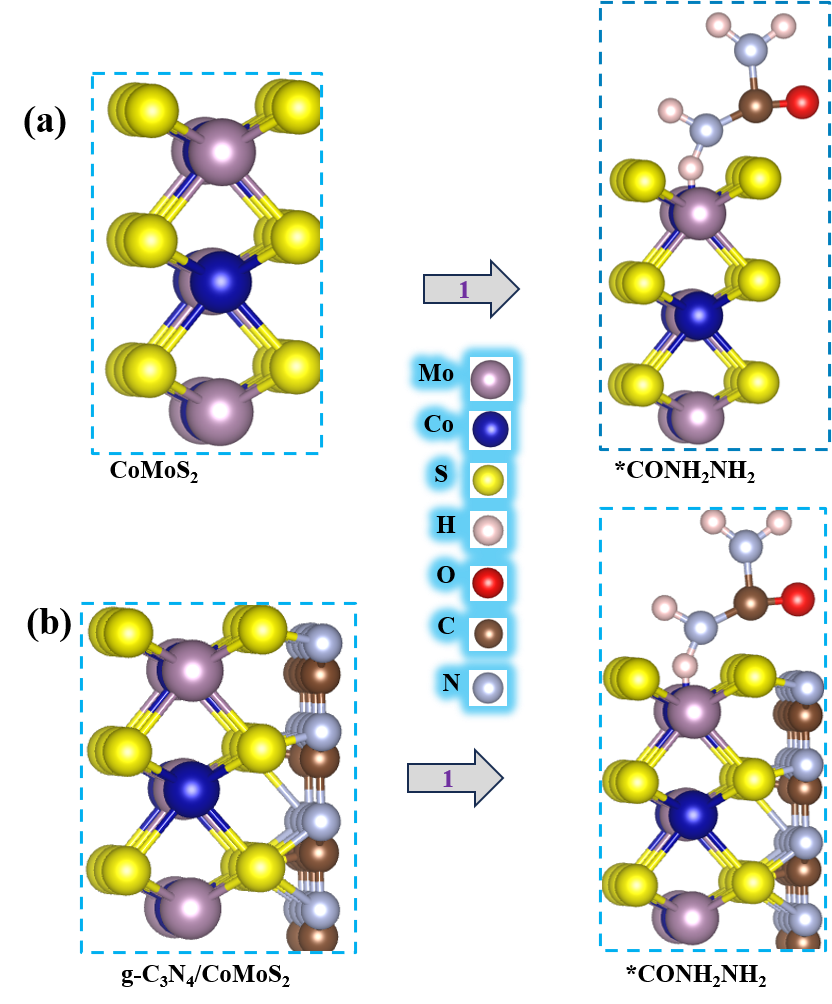


**Figure S18.** Schematic presentation of the atomic structures of urea adsorption on the Co site of a) CoMoS_2_ and b) g-C_3_N_4_/CoMoS_2_


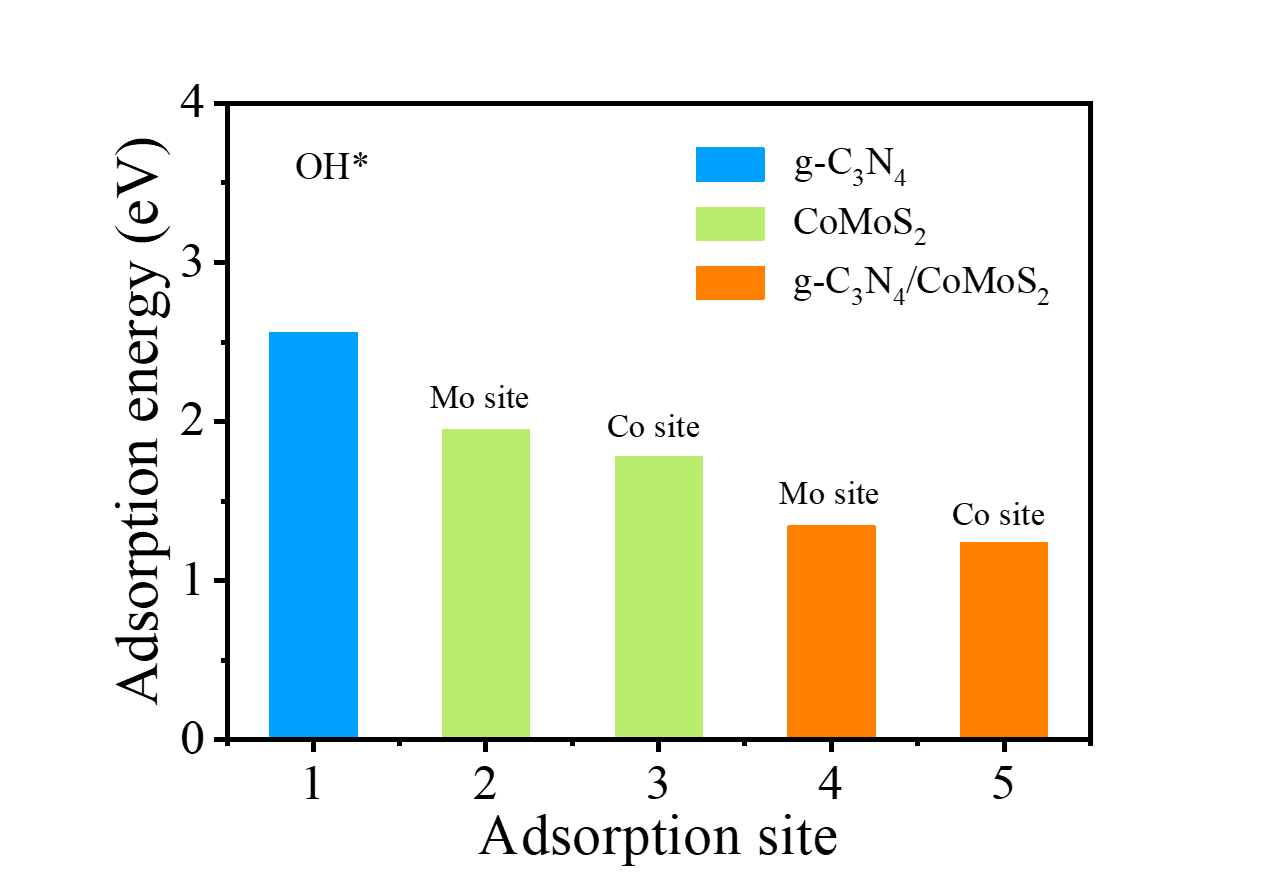
**Figure S19.** Energy profiles for OH * adsorption at different active sites of g-C_3_N_4_, CoMoS_2_, and g-C_3_N_4_/CoMoS_2_ heterojunction.

The calculated OH^-^ adsorption energy of g-C_3_N_4_/CoMoS_2_ (1.24 eV) is markedly lower than that of g-C_3_N_4_ (2.56 eV) and CoMoS_2_ (1.78 eV), suggesting that OH^-^ species preferentially adsorb on the heterojunction surface. This preferential adsorption promotes the dominance of the urea oxidation reaction (UOR) over the oxygen evolution reaction (OER), consistent with the experimental electrochemical behavior. This thermodynamic tendency reflects enhanced urea–surface interactions, thereby facilitating the initial steps of the UOR under alkaline conditions.


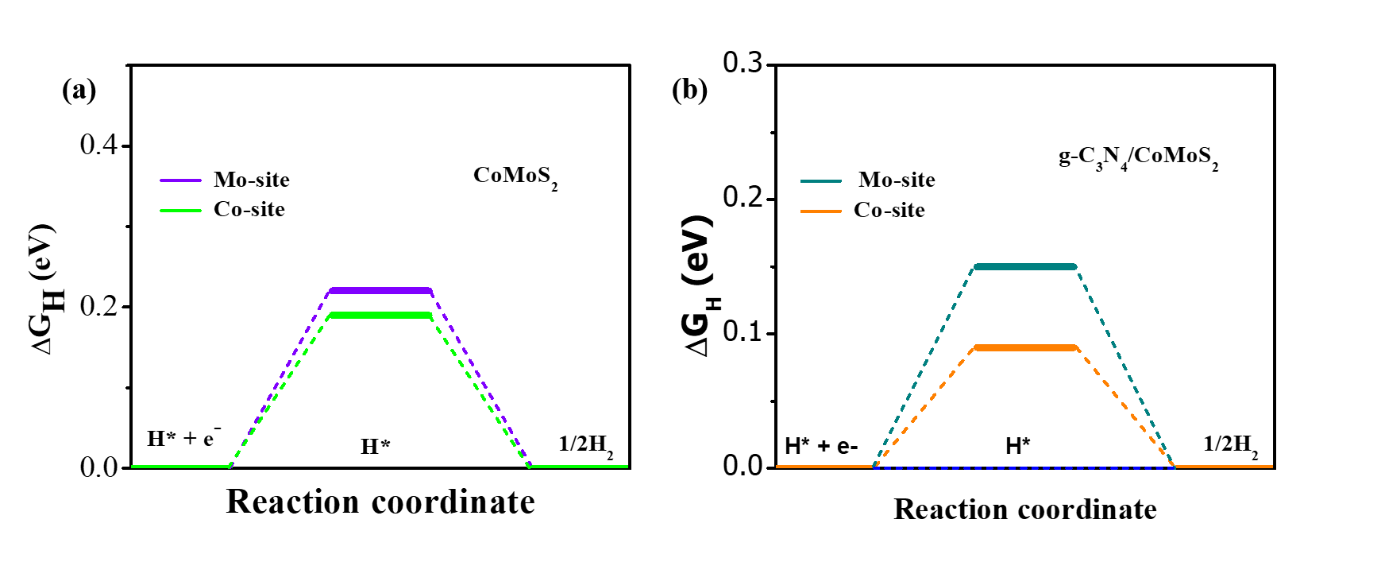


**Figure S20.** Gibbs free energy profiles (*Δ*G_H*_) for hydrogen adsorption at different active sites of a) CoMoS_2_ and b) g-C_3_N_4_/CoMoS_2_ heterojunction.


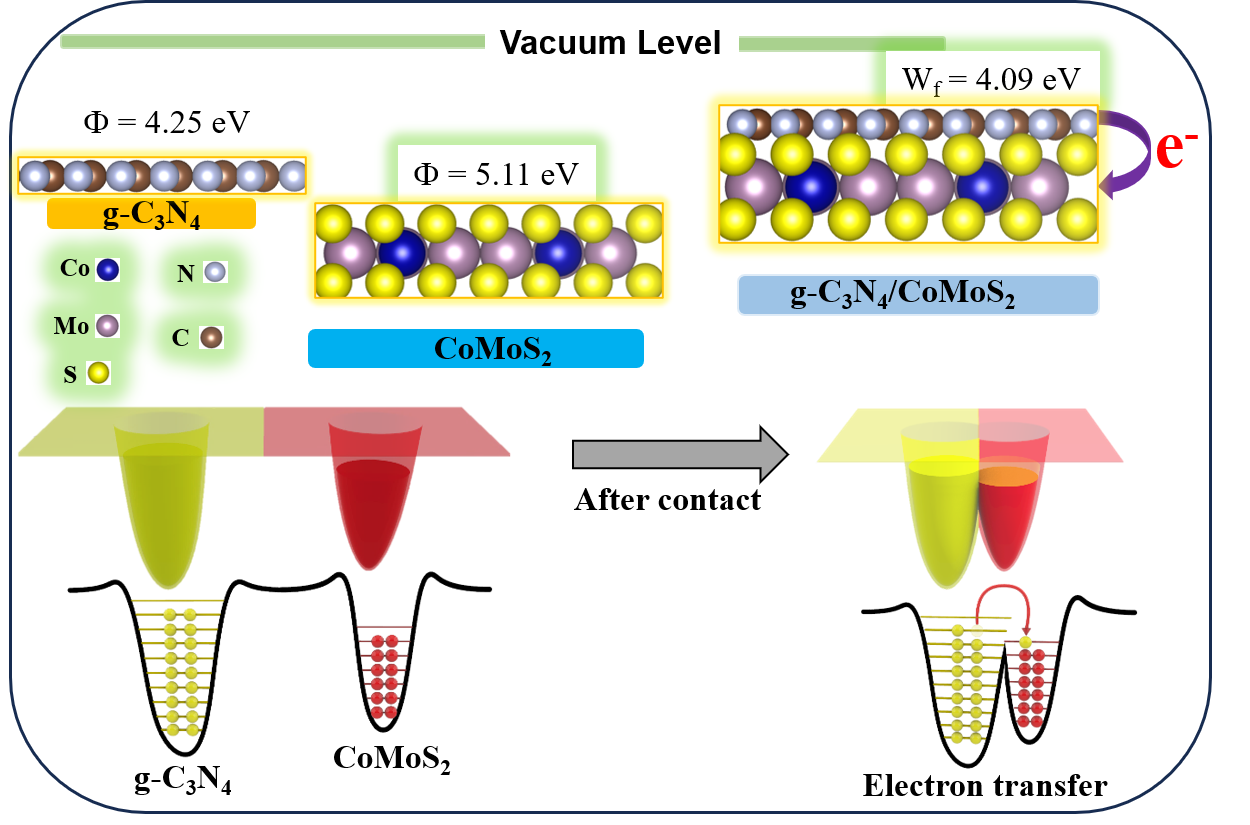


**Figure S21.** Schematic illustration of the electron transfer pathways, highlighting the directional migration of electrons from g-C_3_N_4_ to CoMoS_2_ upon heterojunction formation.


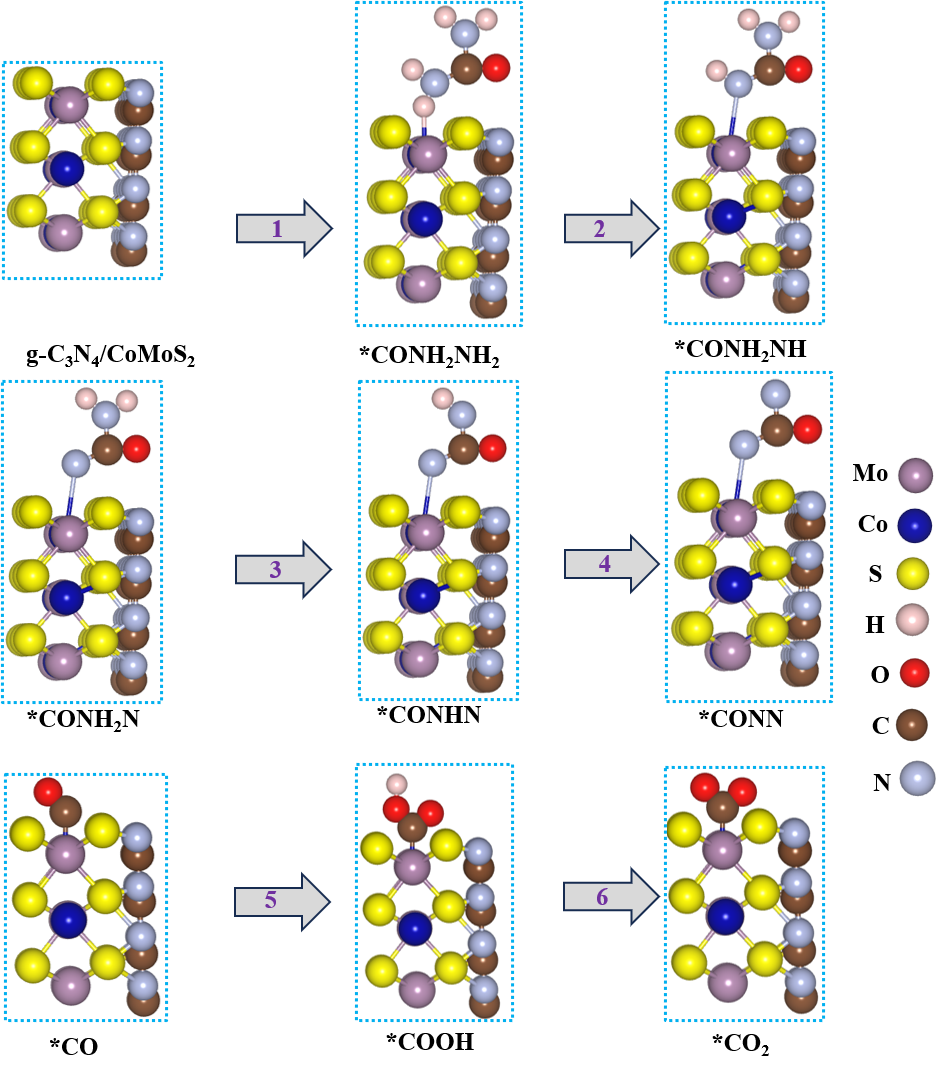


**Figure S22**. Schematic presentation of the atomic structures of intermediate adsorption during the UOR process in g-C_3_N_4_/CoMoS_2_.


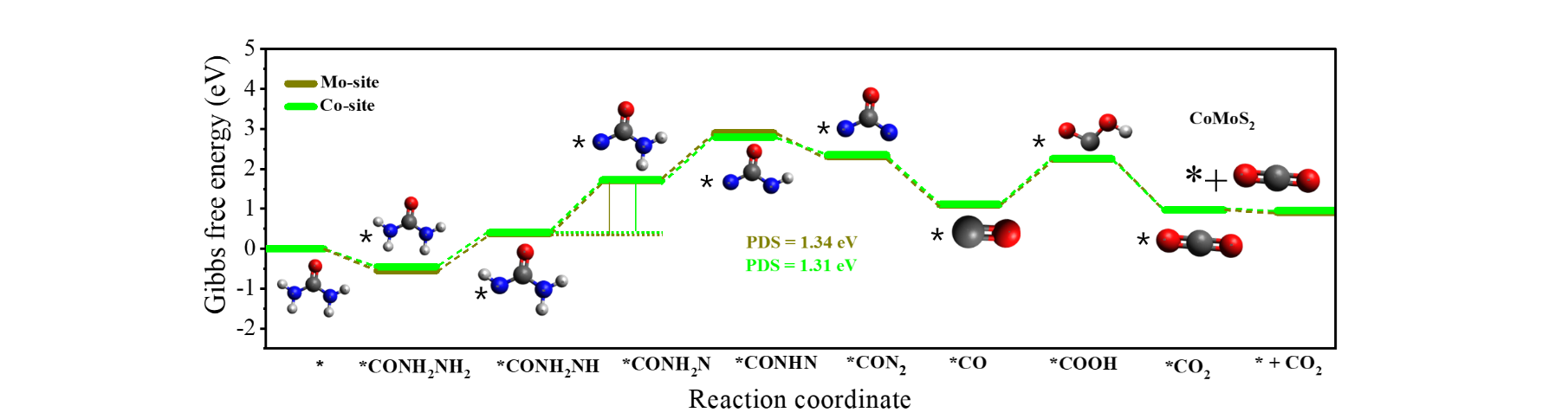


**Figure S23.** Gibbs free energy profiles (*Δ*G) for urea adsorption at different active sites of CoMoS_2_.


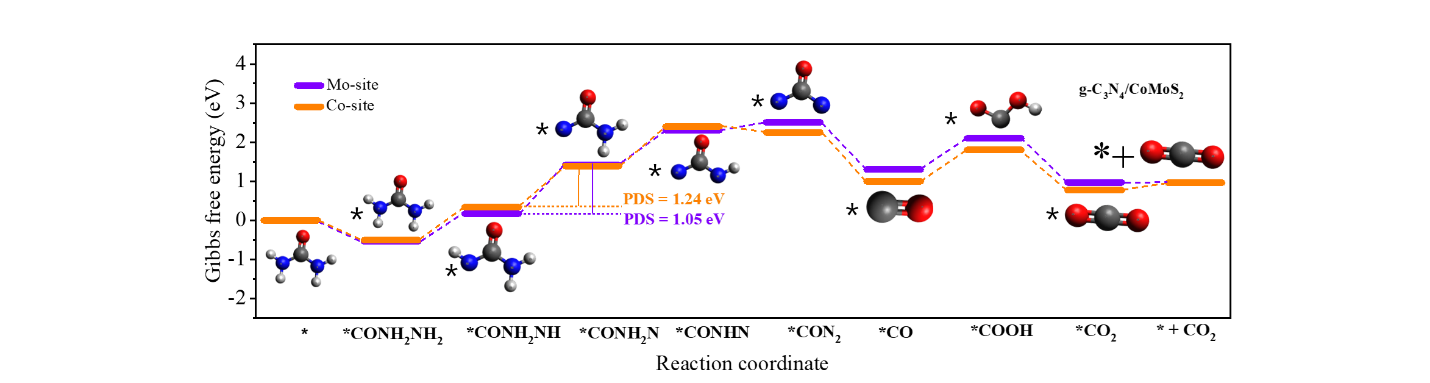


**Figure S24.** Gibbs free energy profiles (*Δ*G) for urea adsorption at different active sites of g-C_3_N_4_/CoMoS_2_ heterojunction.


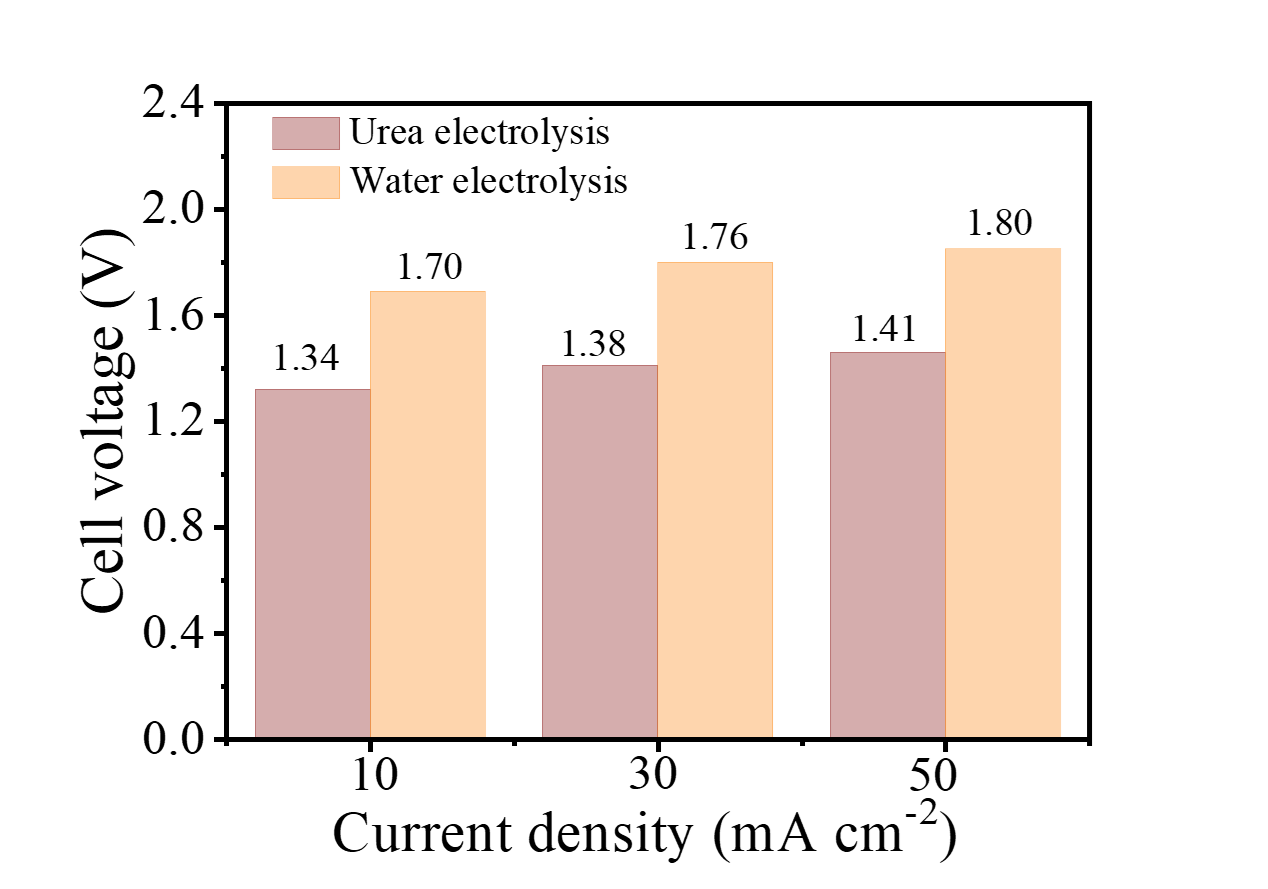


**Figure S25.** Comparison of the corresponding overpotentials at current densities of 10, 30, and 50 mA cm^-2^.


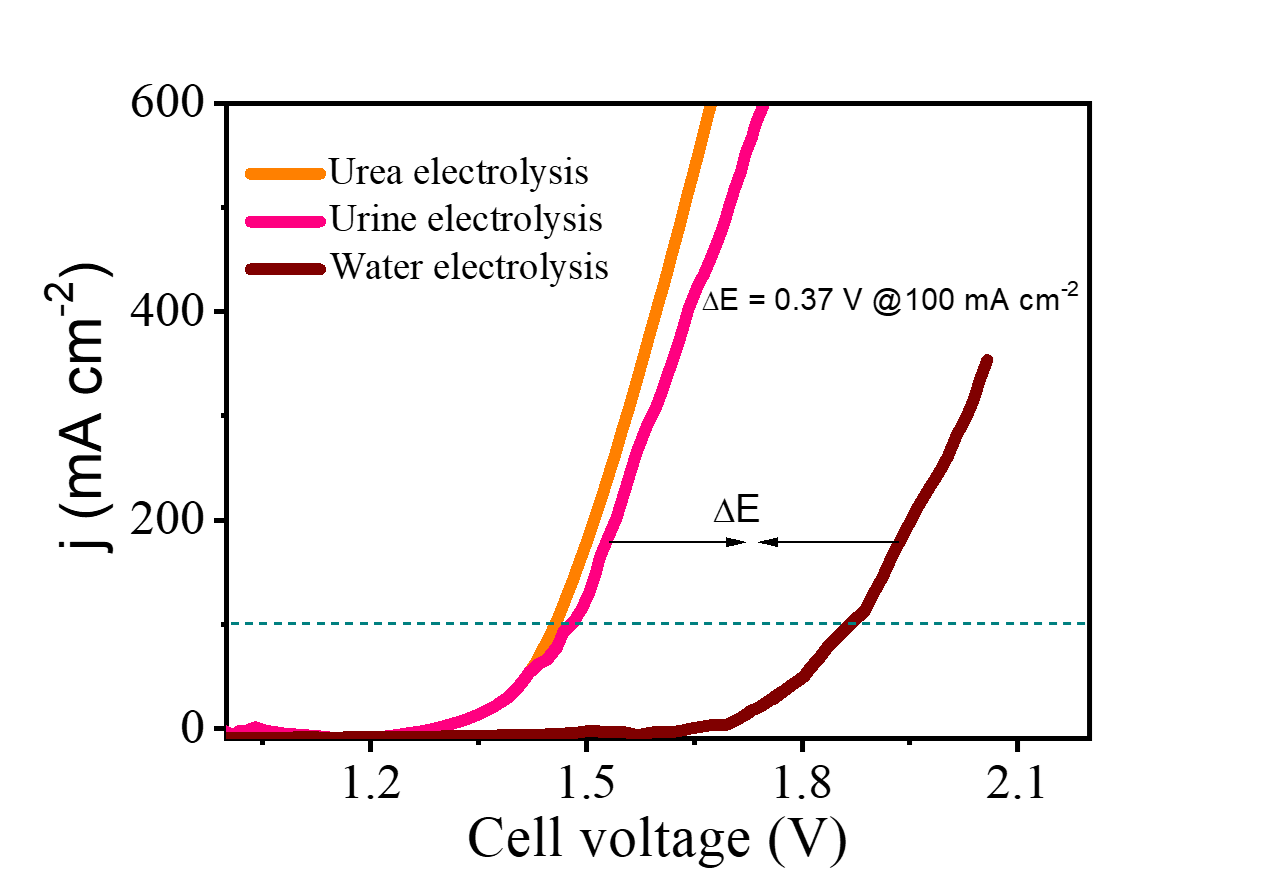


**Figure S26.** Comparison of LSV curves for the g-C_3_N_4_/CoMoS_2_//g-C_3_N_4_/CoMoS_2_ in 1 M KOH, 1 M KOH with 0.33 M urea, and 1 M KOH with human urine.

**Table S2.** Comparison of the HER performance for the g-C_3_N_4_/CoMoS_2_ with other reported in alkaline solutions.

| **Catalysts** | **Electrolyte** | **Overpotential (mV)** | | | **Tafel slope (mV dec^-1^)** | **Stability (h)** | **References** | |  |
| --- | --- | --- | --- | --- | --- | --- | --- | --- | --- |
|  |  | **@10 mA cm^-2^** | **@100 mA cm^-2^** | **@200 mA cm^-2^** |  |  |  |  |  |
| SrRuO_3_/CNT | 1.0 M KOH | 109.0 | - | - | 45.0 ± 3.77 | - | ACS Appl. Energy Mater. 2019, 2, 956−960 | |  |
| CuCo_2_O_4_ | 1.0 M KOH | 115.0 | 217 |  | 153 | 30 | Adv. Mater. Interfaces 2020, 7, 1901515 | |  |
| MoS_x_@NiO | 1.0 M KOH | 406.0 | - | - | 43.0 | 13 | Adv. Funct. Mater. 2019, 29, 1807562 | |  |
| MoS_2_/NiS_2_/CoS_2_ | 1.0 M KOH | 101 | 246 | - | 116 | - | Mater. Today Nano, 2022, 17, 100156 | |  |
| 1D-DRHA MoS_2_ | 1.0 M KOH | 119.0 |  |  | 50.7 | 24 | Appl. Catal. B, 2019, 258, 117964 | |  |
| FeCo/Co_2_P@NPCF | 1.0 M KOH | 260 |  |  | 120 | 1000 cycles | Adv. Energy Mater. 2020, 10, 10, 1903854 | |  |
| H-Fe-CoMoS | 1.0 M KOH | 137 |  |  | 98.0 | 20 | Nano Energy, 2020, 75, 104913 | |  |
| Ir@NG-750 | 1.0 M KOH | 114.0 | - | - | 113 | - | J. Mater. Chem. A, 2020, 8, 19665-19673 | |  |
| MoS_2_/(CoNi@G) | 1.0 M KOH | 150.0 | - | - | 66.0 | 10,000 cycles | Nano energy 2020, 72, 104700 | |  |
| Cu-Ni_3_S_2_ | 1.0 M KOH | 121 | - | - | 86.2 | - | Nanoscale, 2021, 13, 2456-2464 | |  |
| Ni NP/Ni–N–C | 1.0 M KOH | 147.0 | - | - | 114 | - | Energy Environ. Sci., 2019, 12, 149-156. | |  |
| Ni/Mo_2_C‐ NCNFs | 1.0 M KOH | 143.0 | 195 |  | 57.8 | 100 | Adv. Energy Mater. 2019, 9, 1803185 | |  |
| NiCo_2_S_4_@CoMo_2_S_4_ | 1.0 M KOH | 120 | - | - | 37.1 | 72 | *Small*, 2025, 9, 2410848. | |  |
| CoFe(3:1)S_2_/  N-C | 1.0 M KOH | 117 | - | - | 39.0 | - | Small, 2020, 16, 2, 1905075 | |  |
| NiFeP/CC | 1.0 M KOH | 129 | - | - | 76.8 | 300 | Chem. Eng. J. 2021, 420, 129972 | |  |
| MoPS/NC | 1.0 M KOH | 170 | - | - | 50.0 | 10,000 cycles | Appl. Catal., B: Environ, 2019, 245, 656-661 | |  |
| Mo-CoS_2_/NC | 1.0 M KOH | 158 | - | - | 65.0 | 40 | Appl. Surf. Sci., 2023, 623, 157030 | |  |
| **g-C_3_N_4_/CoMoS_2_** | **1.0 M KOH** | **80** | **180** | **210** | **28.38** | **135** | **This work** | | |

**Table S3.** Comparison of the UOR performance for the g-C_3_N_4_/CoMoS_2_ with other reported UOR catalysts in alkaline solutions with urea.

| **Catalysts** | **Electrolyte** | **Potential for urea electrolysis at**  **(**V *vs.* RHE **)** | | | **Stability (h)** | | **References** | |
| --- | --- | --- | --- | --- | --- | --- | --- | --- |
|  |  | **@10 mA cm^-2^** | **@100 mA cm^-2^** | **@200 mA cm^-2^** |  |  |  |  |
| (Fe_0.5_Ni_0.5)0.96_S/Co_9_S_8_/NF | 1.0 M KOH + 0.5 M urea | 1.298 | - | - | 14 | | *Small*, 2025, *21*, 2410987 | |
| Pt–Ni(OH)_2_@Ni-CNFs-2 | 1.0 M KOH + 0.33 M Urea | 1.363 | - | - | - | | Energy Environ. Sci.,2024, 17, 1984–1996 | |
| P-W-NiCo@C | 1.0 M KOH + 0.5 M urea | 1.30 | - | - | - | | Chem. Eur. J., 2024, 484, 149561 | |
| Fe–NiO | 1.0 M KOH + 0.5 M urea | 1.324 | 1.371 |  | 60 | | Inorg. Chem. 2024,63, 17, 7937–7945 | |
| Co_0.5_NiS_2_−Ni_3_S_2_/NF | 1.0 M KOH + 0.5 M urea | 1.33 | - | - | - | | Chem. Eng. 2024, 12, 998−1006 | |
| CF@CoOS-2 | 1.0 M KOH + 0.33 M Urea | 1.36 | - | - | 30 | | Small  2024, 2310112 | |
| Ni_2_P_4_O_12_/NiTe | 1.0 M KOH + 0.33 M Urea | 1.337 | 1.363 | - | 50 | | Adv. Mater. 2024, 2311766 | |
| Ni_2_P–Co_2_P/C | 1.0 M KOH + 0.33 M Urea | 1.27 | 1.32 |  | 200 | | Adv. Funct. Mater. 2023, 33, 2303300 | |
| Ni-WO_3_/NF | 1.0 M KOH + 0.5 M urea | 1.319 | 1.364 | 1.384 | 150 | | Small Methods  2024, 2400108 | |
| f-NiMn-LDH | 1.0 M KOH + 0.33 M Urea | 1.310 | - | - | - | | Chem. Eur. J., 2024 484, 149706 | |
| *β*-Ni(OH)_2_-S/Cu-A | 1.0 M KOH + 0.33 M Urea | 1.30 | - | - | 32 | | Chem. Eur. J., 2024 490, 151251 | |
| **g-C_3_N_4_/CoMoS_2_** | 1.0 M KOH + 0.33 M Urea | **1.27** | **1.33** | **1.41** | **120** | **This work** | |  |

**Reference**

[1] G. Kresse, J. Furthmüller, Physical review B, 54 (1996) 11169.

[2] P.E. Blochl, Phys. rev. B, 50 (1994) 17953-17979.
